# Case Report: Primary Intraosseous Poorly Differentiated Synovial Sarcoma of the Femur

**DOI:** 10.3389/fonc.2022.754131

**Published:** 2022-03-16

**Authors:** Ke Pang, Xiaoning Guo, Yi Jiang, Lina Xu, Lin Ling, Zhihong Li

**Affiliations:** ^1^ Department of Orthopedics, The Second Xiangya Hospital of Central South University, Changsha, China; ^2^ Hunan Key Laboratory of Tumor Models and Individualized Medicine, The Second Xiangya Hospital of Central South University, Changsha, China; ^3^ Department of Pathology, The Second Xiangya Hospital of Central South University, Changsha, China

**Keywords:** synovial sarcoma, bone tumor, small round cell, poorly differentiated, SYT-SSX fusion gene

## Abstract

Primary intraosseous poorly differentiated synovial sarcoma is exceedingly rare. Here, we present a case of primary intraosseous poorly differentiated synovial sarcoma from the proximal femur in a 16-year-old girl. The case was initially misdiagnosed, but the correct diagnosis of synovial sarcoma was eventually confirmed by fluorescence in situ hybridization and next-generation sequencing. We review the literature pertaining to synovial sarcoma and show that this case is the second molecularly proven intraosseous poorly differentiated synovial sarcoma in the literature. Recognition of intraosseous synovial sarcoma composed of small round cells is imperative in order to avoid misdiagnosis of the tumor as Ewing sarcoma and other small round-cell tumors, all of which have markedly different clinical management.

## Introduction

Synovial sarcoma (SS) is an uncommon malignant mesenchymal neoplasm accounting for 5% to 10% of all soft tissue sarcomas (1). It has an annual incidence of 1.348 to 1.548 per 1,000,000 (2). Although SS can affect patients of any age, it most commonly affects young adults between the ages of 15 and 30 years (3–5). Its cell of origin is still a matter of debate, and neural, myogenic, or multipotent mesenchymal stem cells have been considered putative originators. There are three histological classifications: monophasic, biphasic, and poorly differentiated subtypes. In the monophasic variant, the tissue is composed entirely of spindle cells whereas in biphasic synovial sarcoma, there are epithelial and spindle-cell components present. Occasionally, the entire tumor shows poorly differentiated morphology, which resembles other small round-cell neoplasms such as Ewing sarcoma. SS can occur in almost any part of the body. The most common sites for SS are periarticular soft tissue of the lower extremities, particularly the knee; whereas, the bone is a rare location for SS. Primary poorly differentiated intraosseous SS is even rarer.

Due to the rarity of this condition, 13 cases of primary intraosseous SS with molecular confirmation of the diagnosis have been reported so far (6–16). In this paper, we report a rare case of primary poorly differentiated intraosseous SS in a 16-year-old girl. We share the findings observed in the case, putting them in relation with data from the literature and provide some valuable experience in managing difficult cases of small round-cell neoplasms.

## Case Presentation

A 16-year-old girl presented to our hospital with a 7-month history of intermittent episodes of pain in the proximal part of the left thigh. The pain gradually worsened over time, and nocturnal pain occurred. She had no particular notable family history.

On physical examination, she reported a slight pain and tenderness in her left thigh. The swelling or mass was not palpable. The range of motion of the left hip was slightly disturbed. Serum alkaline phosphatase (ALP) and lactic dehydrogenase (LDH) levels were normal.

**Figure 1:**
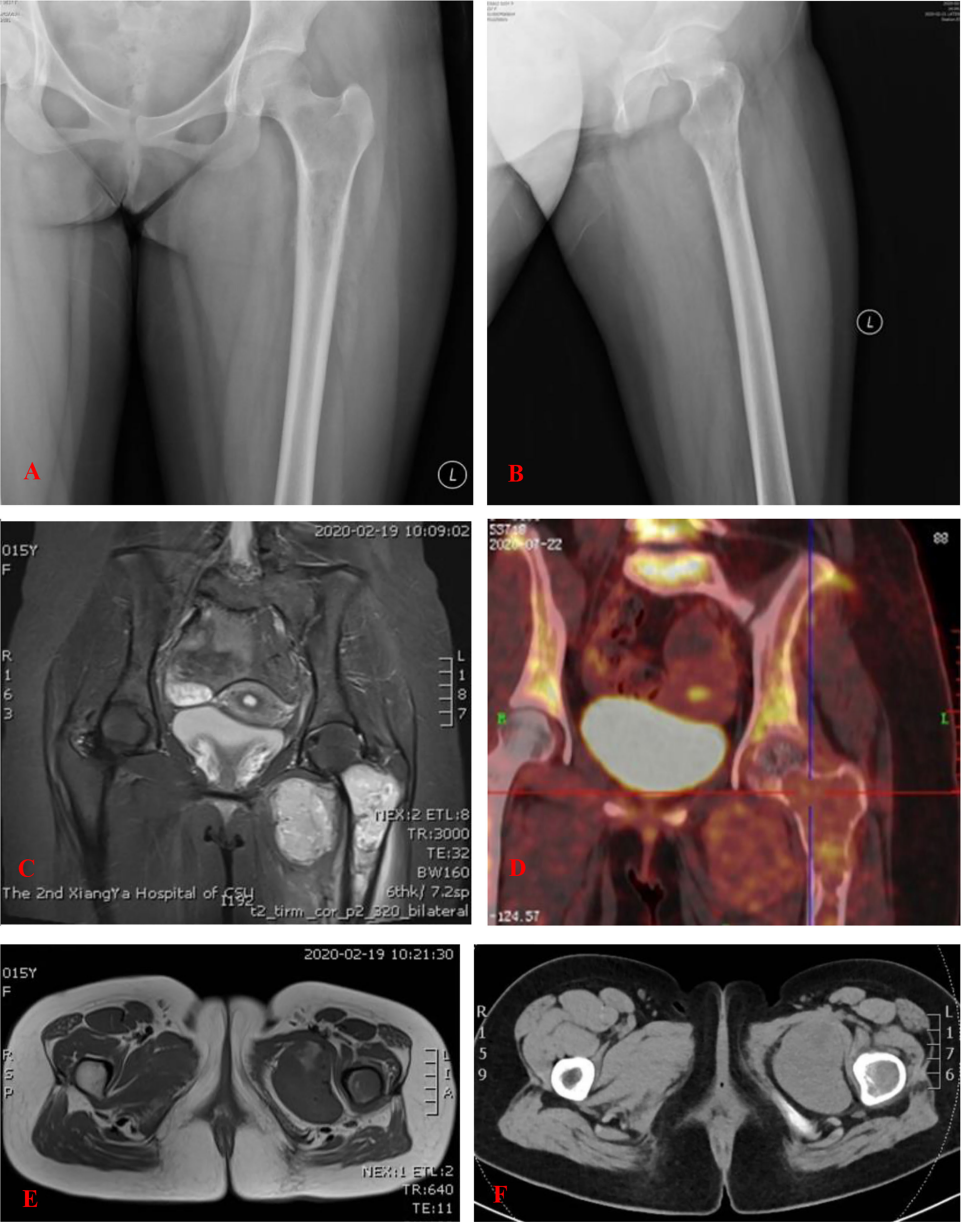
**(A, B)** An anteroposterior and lateral plain radiograph of the left femur. There is a comparatively well-outlined osteolytic lesion at the proximal part of the left femur. **(C, E)** MRI of the lesion of the proximal femur. The mass showed isointensity on T1-WI and high intensity on T2-weighted images (T2-WI). Well-defined oval-shaped heterogeneous soft tissue mass in close proximity to the medial side of the lesser trochanter. **(D)** PET-CT showed slight abnormal fluorodeoxyglucose (FDG) uptake in the lesion. **(F)** CT showed an expansile lesion at the proximal femur in the intertrochanteric region.

**Figure 2:**
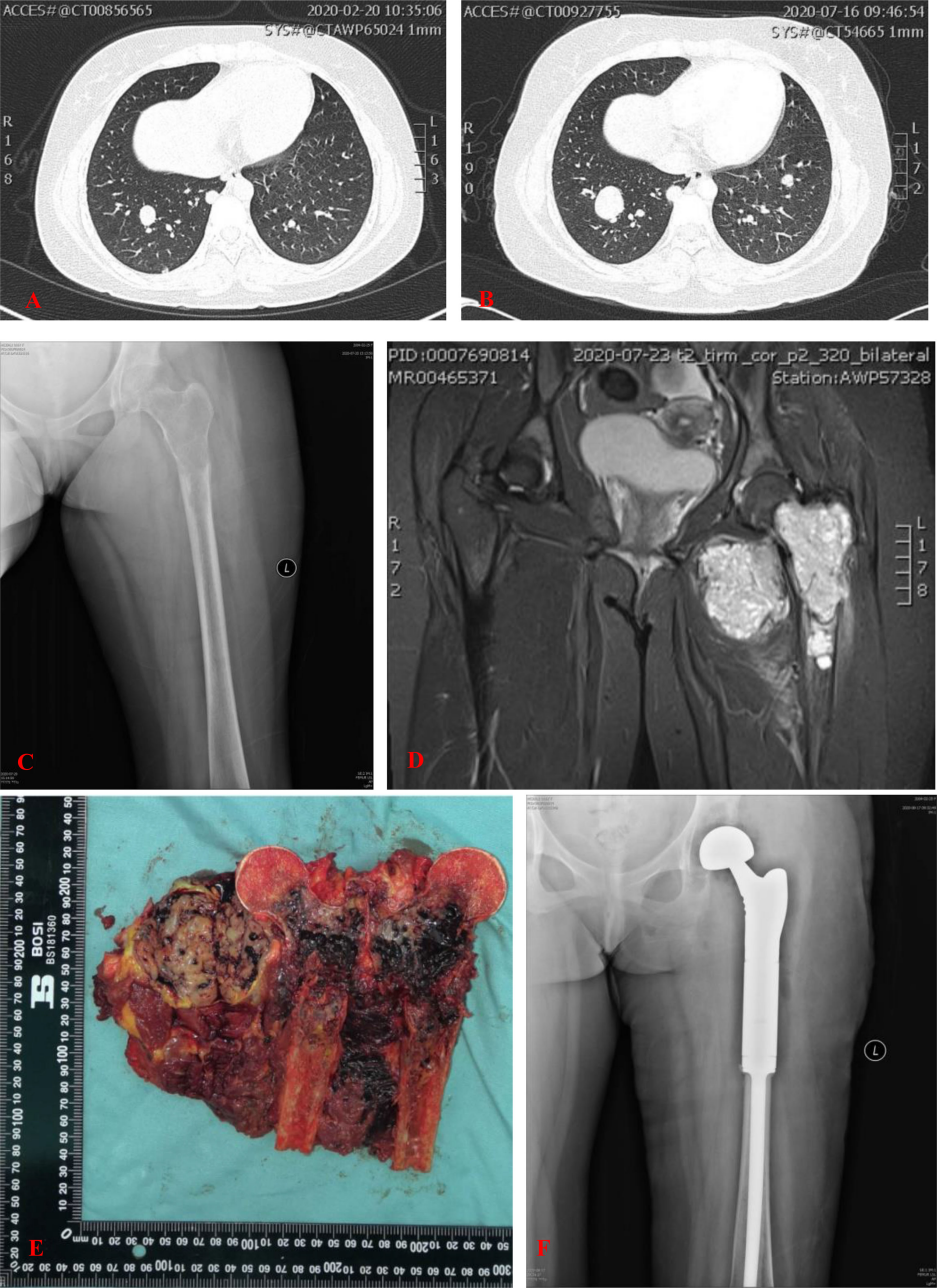
**(A)** CT showed multiple nodules in both lungs. **(B)** CT showed part of nodules in both lungs was enlarged 5 months later. **(C)** Plain radiograph of the left femur showed an aggravated lytic lesion in the upper segment of the left femur. **(D)** MRI showed the intramedullary soft tissue mass was enlarged. Intramuscular soft tissue mass in close proximity to the medial side of the lesser trochanter had no obvious changes. **(E)** Grossly, the tumor was centrically located in the proximal femur. Cut section of the proximal femur showed a black cut surface involving the intramedullary space with areas of hemorrhage and necrosis. The adjacent extraosseous mass had a yellowish-brown fleshy cut surface measured 6.4 × 4.8 cm in axial diameter. **(F)** Postoperative anterior posterior radiographs of the left femur demonstrated the endoprosthetic reconstruction of the proximal femur and hip joint.

X-ray examination revealed a comparatively well-outlined osteolytic lesion in the proximal part of the left femur, and cortical bone around the lesion was thinner, accompanied with periosteal reaction (**Figures 1A, B**). A computed tomography (CT) scan revealed multiple nodules in both lungs (**Figure 2A**) and an expansile lesion at the proximal femur in the intertrochanteric region (**Figure 1F**). Magnetic resonance imaging (MRI) also demonstrated a bone tumor in the proximal part of the left femur. The mass showed isointensity on T1-weighted images and high intensity on T2-weighted images (T2WI) and was heterogeneously enhanced by gadolinium-diethylenetriamine pentaacetic acid (**Figures 1C, E**). Well-defined oval-shaped heterogeneous soft tissue mass in close proximity to the medial side of the lesser trochanter was observed (**Figures 1E, F**). The extraosseous mass measured 6.4 × 4.8 cm in axial diameter and 5.5 × 5.8 cm craniocaudally. SPECT revealed radionuclide concentration in the left superior femur and no abnormal concentration in other bones. Positron emission tomography-CT showed slight abnormal fluorodeoxyglucose uptake in the lesion (**Figure 1D**).

Clinical and imaging findings suggested a malignant bone tumor, but the type of tumor was unknown. Initially, core-needle biopsy was performed and a diagnosis of Ewing sarcoma was considered based on intraoperative frozen section. The results of immunohistochemistry (IHC) and immunofluorescence analyses showed positive expression of CD99 and CD56 and negative expression of S100, NSE, NKX2.2, TTF-1, Myogenin, MyoD1, LCA, SATB2, Syn, CgA, CK, SMA, and Desmin (**Supplementary Materials**). Ki-67 staining indicated a proliferative index of 30%.

Interval-compressed chemotherapy with alternating cycles of vincristine, doxorubicin, cyclophosphamide, ifosfamide, and etoposide (VDC/IE) was recommended as first-line systemic therapy for this patient. She subsequently underwent three cycles of chemotherapy, which consisted of VDC alternating with IE, delivered every 3 weeks. Five months later, reexamination showed that the lytic lesion in the upper segment of the left femur aggravated after three chemotherapy cycles (**Figure 2C**). The intramuscular soft tissue mass in close proximity to the medial side of the lesser trochanter showed no obvious changes (**Figure 2D**). Part of the nodules in both lungs was larger than before (**Figure 2B**).

After completion of the three cycles of chemotherapy, the patient with the American Joint Committee on Cancer (AJCC) stage IVA underwent a wide excision of the tumor at the proximal part of the left femur, including the involved soft tissue (**Figure 2E**), followed by endoprosthetic reconstruction with a bipolar proximal femoral tumor prosthesis (**Figure 2F**).

**Figure 3:**
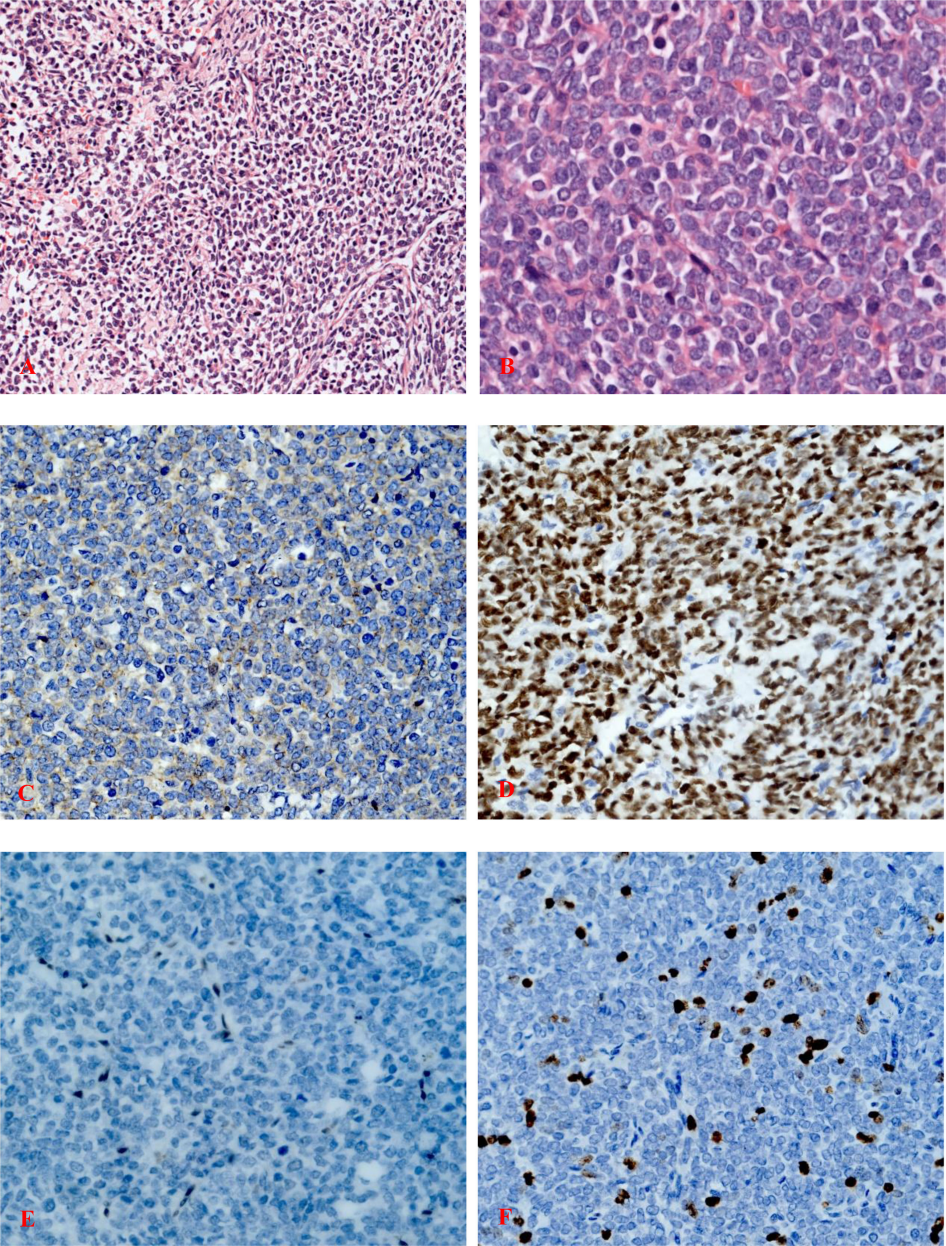
**(A)** Highly cellular, malignant tumor composed of compact sheets of poorly differentiated rounded cells with ovoid or round hyperchromatic nuclei (hematoxylin and eosin staining; magnification, ×200). **(B)** Poorly differentiated round cells with a high nuclear-to-cytoplasmic ratio and vesicular nuclei with nucleoli (hematoxylin and eosin staining; magnification, ×400). **(C)** Immunohistochemistry of tumor cells showing focal positivity for membranous CD99 (magnification, ×400). **(D)** Strong nuclear TLE1 reactivity of the tumor cells (magnification, ×400). **(E)** Immunostaining for FLI-1 was negative (magnification, ×400). **(F)** Ki-67 staining indicated a proliferative index of 15%, ×400.

The tumor occupied the proximal part of the left femur and demonstrated similar characteristics as the specimen obtained from the biopsy. Histopathological examination of the surgically resected specimen revealed a dense distribution of poorly differentiated small round cells in the fibrous tissue (**Figures 3A, B**).

**Figure 4:**
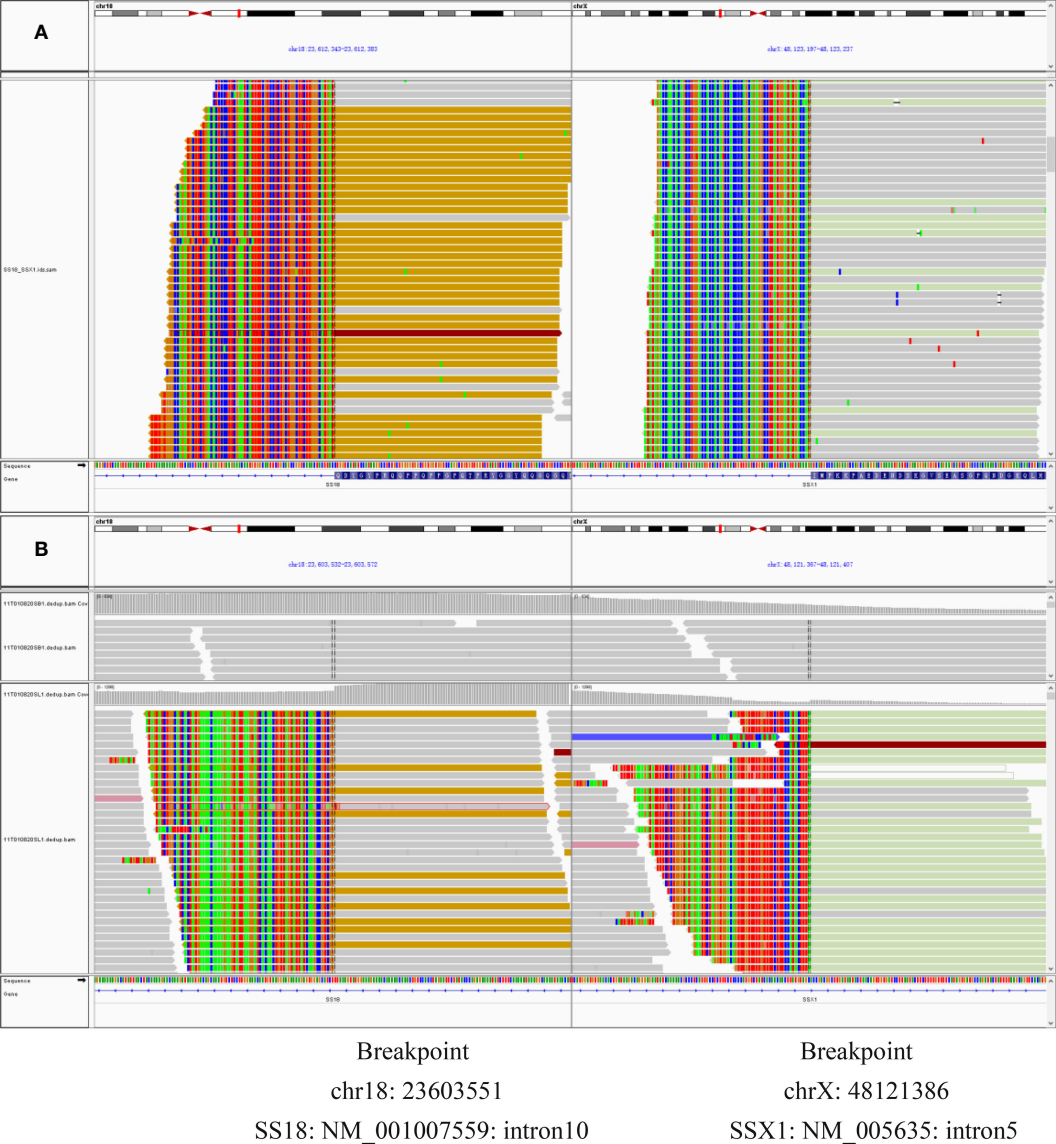
**(A)** RNAseq reads revealed exon 10 of *SS18* was fused in-frame to exon 6 of *SSX1*. **(B)** DNA-seq reads revealed exon 10 of *SS18* was fused in-frame to exon 6 of *SSX1*. Breakpoint was intron 10 of *SS18* and intron 5 of *SSX1*.

IHC staining revealed that the biopsy was positive for CD56, Bcl-2, and TLE1 (**Figure 3D**); focally positive for C99 (**Figure 3C**), CD57, CK, CK8/18, Calponin, and Syn; and negative for Vim, FLI-1, NKX2.2, CgA, S100, NSE, LCA, Desmin, WT-1, SATB2, PD-1, and PD-L1 (**Supplementary Materials**). Ki-67 staining indicated a proliferative index of 15% (**Figure 3F**). Fluorescence in situ hybridization (FISH) analysis for EWSR1-split was negative but that for SS18-split was positive. This result provided the definitive diagnosis of SS. High-throughput next-generation sequencing (NGS) further revealed a chimeric SS18 exon 10-SSX1 exon 6 transcript (**Figure 4**); these findings were consistent with SS.

Postoperative CT showed that multiple metastatic lesions in both lungs were larger than before. Considering the progress of the disease and the fact that a variety of chemotherapy drugs had been used before but chemotherapeutic efficacy was not obvious, the patient underwent chemotherapy with docetaxel and gemcitabine.

**Figure 5:**
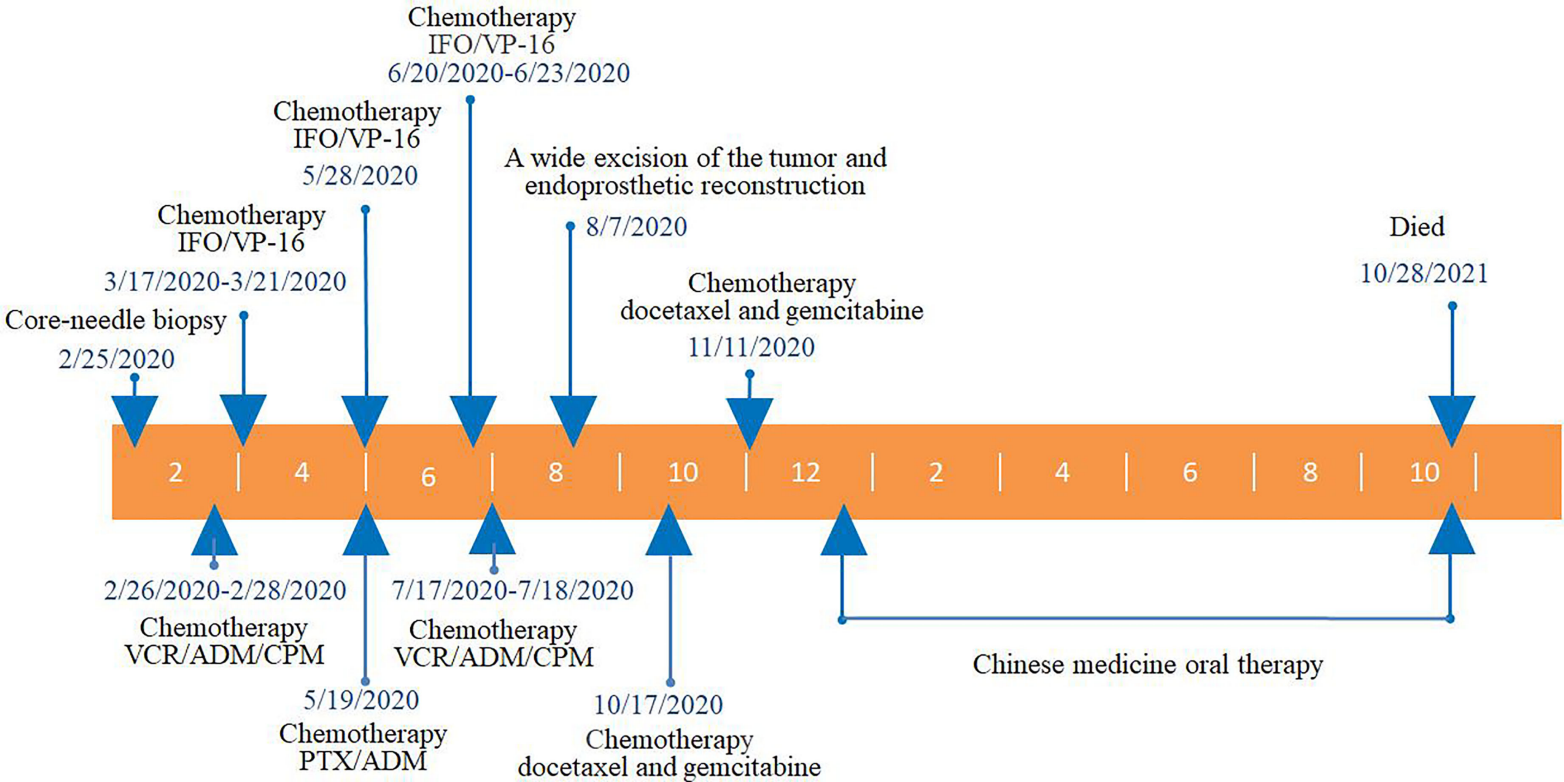
The whole clinical process of this patient. VCR, vincristine; ADM, adriamycin; CPM, cis-platinum; PTX, paclitaxel; IFO, ifosfamide; VP-16, etoposide.

At the 14-month follow-up, the patient declared that low back pain had developed 10 months after surgery, which was considered to be caused by lumbar spine metastasis, and death occurred 14 months after surgery almost immediately after the last follow-up. The whole clinical process of this patient is shown in **Figure 5**.

## Discussion

SS is a malignant mesenchymal neoplasm most commonly arising in the deep soft tissues of the extremities in adolescents and young adults. SS can occur in almost any anatomic site. It arises more often from the soft tissue near joint spaces and very rarely presents as a primary bone tumor. To the best of our knowledge, this case is only the second molecularly proven poorly differentiated intraosseous SS and the first reported case of intraosseous SS arising from the femur in the literature (**Table 1**).

**Table 1:** Summary of all reported cases of primary intraosseous synovial sarcoma with molecular confirmation of the diagnosis.

Year	Author	Age	Sex	Location	Morphology	IHC	Confirmation
2021	Pang et al. (current study)	16	F	Femur	PD	CD56(+), Bcl-2(+), TLE(+), C99(focal+), CD57(focal+), CK(focal+), Syn(focal+), CK8/18(focal+), Calponin(focal+), Vim(−), FLI-1(−), CgA(−), S100(−), NSE(−), LCA(−), Desmin(−), NKX2.2(−), WT-1(−), SATB2(−), PD-1(−), PD-L1(−)	*SS18::SSX1* fusion

2020	McHugh et al.	45	F	Humerus	Monophasic	CD99(+), EMA(focal+), STAT6(−), AE1/3(−), CD34(−)	FISH
2020	McHugh et al.	36	M	Metatarsal	Monophasic	CD99(+), STAT6(−), CD34(−), CK20(−), SMA(−), S100−),CAM5.2(−), Melan A(−), Desmin(−), pankeratin(−)	FISH
2019	Caracciolo et al.	33	M	Femur	Monophasic	CKAE1/3CAM(+), CK7(+), CK8/18(+), CD99(+), EMA(weakly+), Bcl-2(+)	*SS18::SSX1* fusion
2019	Horvai et al.	33	M	Tibia	Monophasic	Keratin(+), EMA(+), INI1(+), S-100(−), TLE1(−)	*SS18::SSX2* fusion
2019	Horvai et al.	36	M	Tibia	Monophasic	Keratin(+), EMA(+), TLE1(+), INI1(+), p63(+), S-100(−CK5/6(−), Bcl-2(+), CD34(−)	*SS18::SSX1* fusion
2019	Fujibuchi et al.	77	F	Ulna	Monophasic	Bcl-2(+), EMA(focal+)	*SS18::SSX1* fusion
2014	Cao et al.	26	M	Thoracic spine	Biphasic	CD68(+), CD34(+), VIM(+), Bcl-2(+), CD56(+), CKpan(−), SMA(−), DES(−), S100(−)	FISH
2011	Beck et al.	53	M	Tibia	Biphasic	EMA(+), keratin(+), cytokeratin 7(+)	FISH
2010	Verbeke et al.	73	F	Fibula	Monophasic	N/A	FISH
2007	Jung et al.	21	F	Tibia	Monophasic	Cytokeratin(+), EMA(+), Bcl-2(+), vimentin(+)	*SS18::SSX1* fusion
2006	O’Donnell et al.	37	M	Ulna	PD	EMA(+), CD99(+), Bcl-2(−), S100(−), SMA(−), Desmin(−)	*SS18::SSX1* fusion
1999	Hiraga et al.	67	M	Radius	Monophasic	EMA(+), CK(−)	RT-PCR
1997	Cohen et al.	22	M	Tibia	N/A	MIC-2 (+), keratin(+), synaptophysin(+), vimentin(+), S-100(focal+) EMA(−), α-SMA(−), HHF-35(−), Desmin (−), LCA(−), Leu-7(−), PAS(−)	Spectral karyotyping

M, male; F, female; PD, poorly differentiated; FISH, fluorescent in situ hybridization; IHC, immunohistochemical; N/A, not available.

Diagnosing biphasic SS is generally straightforward, owing to distinctive histologic features. However, the differential diagnosis of monophasic and poorly differentiated SS may be more challenging. The diagnosis of entirely poorly differentiated SS, which is often confused histologically with other small round-cell tumors, can be more difficult. In addition, intraosseous SS further increases the difficulty of diagnosis.

In our case, the histopathological examination revealed a dense distribution of small round cell in fibrous tissue. Based on cell morphology and IHC results, the initial diagnosis was Ewing sarcoma. The pathologists believe that the misdiagnosis of Ewing sarcoma is mainly due to the positive expression of CD99 and CD56 in small round cells. High CD99 expression has been shown in Ewing sarcoma, hence it was initially believed to be a specific marker for Ewing sarcoma (17) and routinely used for the differential diagnosis of Ewing sarcoma from other types of small round-cell tumors.

CD99, although positive in 90% (18–20) of Ewing sarcomas, lacks specificity since it is also expressed in other mesenchymal and lymphoid neoplasms, including frequent expression in SS (21, 22). As a result, it is important to be aware of CD99 positivity in SS since it can resemble a small round-cell tumor and be misdiagnosed as Ewing sarcoma with a limited antibody panel.

CD56, commonly considered natural killer cell and neuroectodermal markers (23), has been identified in neuroendocrine neoplasms and some soft tissue and bone tumors, including undifferentiated small-round blue cell tumors (24–26). Besides neural cell adhesion molecules (CD56), other neuroendocrine markers synaptophysin (Syn), neuron-specific enolase (NSE), and chromogranin A (CgA) are often used in differential diagnosis of sarcoma. In our case, IHC staining of biopsy samples disclosed negativity for Syn, CgA, and NSE and only CD56 was positive. It is true that CgA and Syn expression is very limited among small round-cell tumors, being confined to esthesioneuroblastomas, differentiating neuroblastomas, and small cell carcinomas, including Merkel cell carcinoma (27). However, CD56 may be expressed by a variety of sarcomas, including SS, malignant peripheral nerve sheath tumors, leiomyosarcoma, chondrosarcoma, and osteosarcoma. Hence expression of CD56 is nonspecific for Ewing sarcoma.

Besides the aforementioned diagnostic IHC panel of antibodies, FLI-1 protein expression is also helpful in distinguishing Ewing sarcoma/primitive neuroectodermal tumor (EWS/PNET) from other tumors that may be CD99 positive, such as poorly differentiated SS and rhabdomyosarcoma. The sensitivity and specificity of FLI-1 to distinguish Ewing sarcoma/primitive neuroectodermal tumor from other small round-cell tumors are 74.2% and 91.6%, respectively (28). In our case, CD99 expression of biopsy specimens is positive. However, the negative staining for FLI-1 argued against a diagnosis of a classical ES (17, 29) (**Figure 3E**). IHC detection of FLI-1 is more specific for Ewing sarcoma than is CD99. FLI-1 is still a highly specific marker to distinguish EWS/PNET from all types of malignancies. In addition, in poorly differentiated SS, alveolar rhabdomyosarcoma, mesenchymal chondrosarcoma, and other tumors with positive CD99 expression, FLI-1 is negative (28, 30, 31). That is exactly what happened in this case; CD99 was positive while FLI-1 was negative. This helped us to make a diagnosis of SS.

It is a pity that TLE1 was not initially detected in the biopsy specimens. TLE1 is most commonly considered in the differential diagnosis as a diagnostic immunomarker for distinguishing SS from tumors. Chin et al. (32) found that 82% of SS were positive for TLE1, including 79% of monophasic type, 78% of biphasic type, and 91% of poorly differentiated type SS. The positive rate of TLE1 in poorly differentiated SS is relatively high. In this case, TLE1 was detected positive in the surgical resection specimen, which further supported the diagnosis of SS. TLE1, while a sensitive marker for SS, is not specific, with expression not infrequently being reported in other tumors including potential mimics. TLE1 expression can be heterogeneous (33) and has also been reported in up to one-third of non-SS cases, including those in its histologic differential diagnosis (34). Of the latter, 15% to 30% of malignant peripheral nerve sheath tumors (MPNST) and 8% of solitary fibrous tumor (SFT) have shown TLE1 expression (although most only weakly) (32, 33). TLE1 alone is not sufficient for the diagnosis of SS since it is present in other tumors, particularly MPNST and SFT.

When encountering a solitary primary malignant small round-cell tumor of the bone, the differential diagnosis generally includes the Ewing sarcoma family of tumors, small cell osteosarcoma, mesenchymal chondrosarcoma, and lymphoma; exceptionally, SS, rhabdomyosarcoma, and desmoplastic round-cell tumors can occur as primary bone tumors (35–37).

To distinguish small cell osteosarcoma from other primary small cell malignancies of bone, Righi et al. (38) evaluated the IHC expression of CD99 and SATB2 in 36 cases of primitive small cell osteosarcoma of the bone. All stained cases were positive for SATB2 expression, which is believed to provide one of the key diagnostic clues to distinguish small cell osteosarcomas from other small round-cell malignancies of the bone.

Microscopically, mesenchymal chondrosarcoma shows a biphasic pattern made up of solid areas of round or spindle mesenchymal cells interspersed with islands of well-differentiated cartilage. It has been proposed that IHC positivity for collagen II and IIA, which are considered to be markers of chondroprogenitor cells, could be used to differentiate mesenchymal chondrosarcoma from other small round-cell malignancies, such as small cell osteosarcoma and Ewing sarcoma (39).

Rhabdomyosarcoma and desmoplastic round-cell tumors are primarily soft tissue neoplasms; rare primary bone tumors are cited in the literature as case reports (36, 37). Myogenin and MyoD1 are expressed in normal fetal skeletal muscle but expression is infrequent in mature muscle (40, 41). Thus, immunostaining for these two proteins is highly specific for rhabdomyosarcomas. Almost all rhabdomyosarcoma samples show positive nuclear staining with antibodies to MyoD1 and/or myogenin, with nonrhabdomyosarcoma pediatric tumors being consistently negative (42). CD99, being negative in more than 50% of cases, is also not specific marker for RMS (43, 44). Desmoplastic small round-cell tumors (DSRCTs) possess a distinctive, diagnostically significant immunophenotype, showing coexpression of epithelial (keratin), mesenchymal (desmin, vimentin), and neural (NSE) markers.

Primary lymphoma of the bones is a rare malignancy. It most commonly affects middle-aged to elderly patients. TdT is the most specific and sensitive marker of lymphoblastic lymphoma/leukemia, with a positive diagnosis rate of 95%, which can be expressed by T and B lymphoblasts. CD34, CD99, and CD43 are also sensitive markers for the diagnosis of lymphoblastic lymphoma/leukemia.

To date, no single IHC marker or combination of markers can definitively confirm or exclude the diagnosis of SS. Highly sensitive and specific IHC markers, particularly ones that would replace the need for FISH studies or molecular testing in routine clinical practice would therefore be highly beneficial. Baranov and his colleagues (45) more recently developed two novel antibodies to detect the SS18::SSX fusion protein: A SS18::SSX fusion-specific antibody (E9X9V clone) that binds to amino acid residues surrounding the SS18::SSX fusion site and a SSX-specific antibody (E5A2C clone) that binds to the C-terminus of the SSX protein. The study of Baranov et al. demonstrated that a novel SS18::SSX fusion-specific antibody is highly sensitive and specific in the diagnosis of synovial sarcomas when used in IHC, with the SS18::SSX antibody having a sensitivity of 95% and specificity of 100% and an antibody to the SSX C-terminus is also highly sensitive but slightly less specific, the SSX C-terminus antibody exhibiting a sensitivity of 100% and specificity of 96%. In a subsequent study, Zaborowski et al. (46) found that the SS18::SSX IHC have 87% sensitivity and 100% specificity, while the SSX C-terminus IHC had 92% sensitivity and 93% specificity. A simple principle for definitive diagnosis of SS was proposed as follows: if a tumor with compatible morphology is SS18::SSX IHC positive then the diagnosis of SS is definitively confirmed and no further testing is required. If a tumor is SS18::SSX IHC negative but SSX C-terminus IHC positive the diagnosis of SS is possible but not confirmed and ancillary testing is recommended. If a tumor is negative for both SS18::SSX and SSX C-terminus IHC, then the diagnosis of SS virtually excludes and does not proceed to FISH or molecular testing unless the morphology was very typical and/or there were confounding factors. If their results are supported by multicenter data, SS18::SSX IHC appears to correlate better with NGS than FISH and seems to have the potential to be transformative and replace the need for FISH studies or molecular testing in most cases of SS.

SS is uniquely characterized by balanced chromosomal translocation, demonstrable in virtually all cases (47), not found in any other human neoplasms. SS is marked by the presence of a pathognomonic translocation between chromosomes X and 18, t (X;18) (p11.2;q11.2), which translates into the expression of several different SS18::SSX fusion proteins, among which the most common are SS18::SSX1, SS18::SSX2, and, much more rarely, SS18::SSX4. The fusion is detectable in 95% of patients and represents an important diagnostic tool (48). In addition, Kao et al. (49) reported an unusual case of intraneural SS harboring an SS18L1::SSX1 fusion.

**Figure 6:**
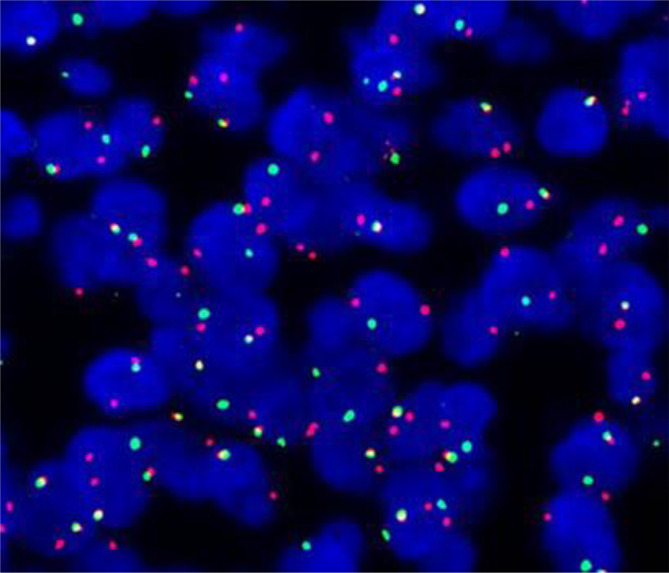
Positive fluorescence *in situ* hybridization (FISH) analysis for *SYT* (*SS18*) gene rearrangement, demonstrated by an abnormal signal pattern seen as disruption of the *SYT* gene through the breaking apart of the red and green probe signals.

*SS18*::*SSX* fusion-specific antibody and *SSX* C-terminal antibody were not widely available at that time, so the two novel antibodies were not used for the diagnosis of this case. Based on the importance of molecular testing, FISH and high-throughput next-generation sequencing were performed on a surgical resection specimen. EWSR1 gene rearrangements were not detected in this case. FISH successfully detected a rearrangement involving the SYT gene (**Figure 6**). Additionally, DNA sequencing and RNA sequencing further revealed exon 10 of SS18 was fused in-frame to exon 6 of SSX1 (**Figure 4**). The breakpoint was intron 10 of SS18 and intron 5 of SSX1 (**Figure 4**). This was eventually diagnosed as SS.

**Figure 7:**
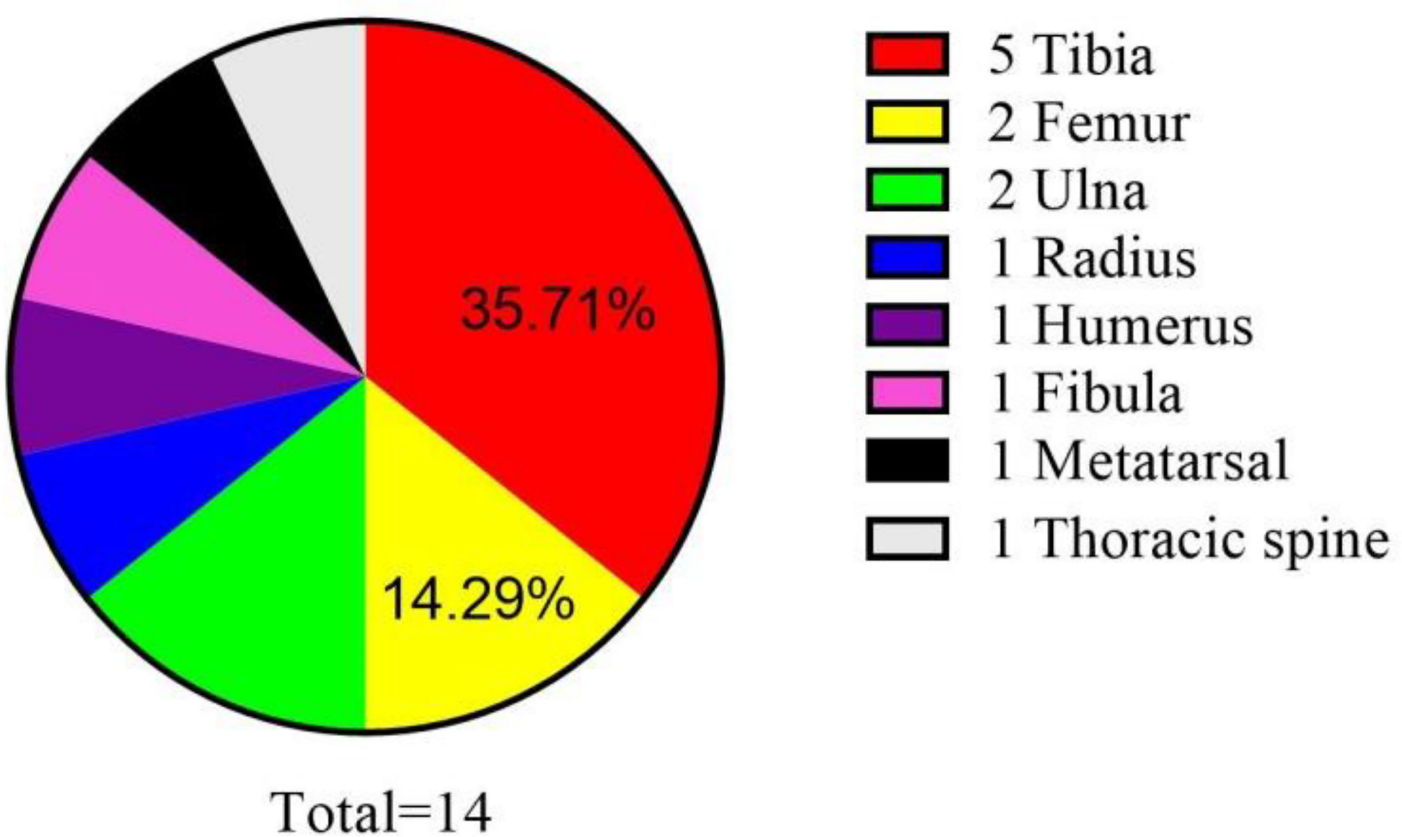
Reported cases of primary intraosseous synovial sarcoma with molecular confirmation of the diagnosis.

Intraosseous SS was first reported in 1997 (6). There have been only 14 molecularly proven cases of SS arising from the bone (6–16). Osseous sites reported include the tibia, femur, ulna, radius, humerus, fibula, and metatarsal and thoracic spine (**Table 1**; **Figure 7**). In **Table 1**, we can see that there is a slight male preponderance, and most patients are over 20–40 years of age. Most IHC markers are not specific, but most of the cases were at least focally positive for CD99 and EMA. The most common sites for intraosseous SS are bone of the lower extremities and the most common of SS18::SSX fusion proteins are SS18::SSX1.

We believe this case represents a primary bone tumor because the center of the lesion is intraosseous, located in the center of the marrow cavity. The proximal femur lesion appeared almost entirely intramedullary. The soft tissue mass was involved medially, but the medial lesion of bone was not worse than the lateral side. Soft tissue involvement was not connected to intraosseous lesions in close proximity to each other. It was thought that the soft tissue tumor was an outward invasion of the bone tumor. We did not consider primary soft tissue tumor metastasis to bone, because no other bone metastases were discovered. Osseous metastasis in soft tissue malignancy is characterized by multiple osteolytic lesions. In addition, patients with metastatic malignant tumor tend to be older than those with SS and often have a history of previous or primary malignant tumor. Moreover, there is a small possibility of metastasis from the soft tissue tumor to the entire cross-section of the femur in a short time. Five months after chemotherapy, the lytic lesion in the upper segment of the left femur aggravated, while the intramuscular soft tissue mass in close proximity to the medial side of the lesser trochanter had no obvious changes.

Intraosseous SS presents as nonspecific, osteolytic destruction of the bone with an unclear boundary. Intraosseous SS, characterized by osteolytic destruction of bone and formation of a soft tissue mass, shows a heterogeneous signal. Heterogeneity is caused by areas of necrosis, calcification, cysts, and hemorrhage within the tumor (50). The imaging features suggestive of SS are characterized by the presence of periarticular, multiloculated, or lobulated cystic masses with heterogeneous septal and/or peripheral nodular enhancement near a large joint. Calcification and bone erosions can be observed (51, 52). Jones et al. (53) described a “triple signal” pattern on T2-WIs, with areas that are of high, intermediate, and low signal intensity relative to fat, thought to reflect a mixture of cystic, hemorrhagic, and solid/fibrous components. The “triple sign” in the tumor was pathologically confirmed as a high signal in fresh hemorrhage and necrotic areas, a medium signal in solid parts of the tumor, and a low signal in fibrous septum and old hemorrhage within the tumor. As we can see, the cross-section of the proximal femur showed a black surface involving the intramedullary space with hemorrhagic foci (**Figure 2E**).

Magnetic resonance imaging of Ewing sarcoma of bone reveals marrow replacement (100%) and cortical destruction (92%), with an associated soft tissue mass in 96% of cases (54–58). The soft tissue mass is commonly circumferential but asymmetric about the osseous involvement (59). However, in our case, the soft tissue mass is on the side of tumor lesion (**Figure 1**). Therefore, the diagnosis of Ewing sarcoma is not supported.

Accurate diagnosis is important as SS is moderately to highly sensitive to chemotherapy with agents such as ifosfamide and anthracyclines. However, the patient died 14 months after surgery. As previously reported, poorly differentiated cases behave more aggressively and metastasize more frequently (60). In this case, there were bilateral pulmonary metastases at the time of diagnosis. The poor prognosis in the case reported here was attributed to poor sensitivity to chemotherapy and early pulmonary metastasis. Nevertheless, in the only previously reported case of poorly differentiated intraosseous SS, the patient remained well with no sign of local or distant disease 1 year following surgery (8). From our point of view, the main cause may be the absence of preoperative pulmonary metastasis and the importance of amputation. Therefore, the revised diagnosis is beneficial for the patient and doctors to know disease and prognosis.

Due to their rarity and heterogeneity, the accuracy of sarcoma diagnosis remains challenging. In the diagnosis of sarcomas, tumor cell morphology and the expression of differentiation markers represent the most important factors, but molecular investigations are increasingly employed to complement these pathological assessments. Commonly, FISH or RT-PCR are used to detect fusion events at the genomic or transcriptional level, respectively. However, both methods present limitations. NGS has laid down the bases to overcome this limitation. By allowing the simultaneous analysis of a large set of targets, NGS, as a support in the case of suspicious diagnosis, has disclosed the possibility not only to reveal diagnostic/prognostic/predictive genetic abnormalities in the absence of a prior hypothesis but also to identify new aberrations (61).

The study presents several limitations. First, due to the rarity of the investigated disease, only one patient was involved in the study. Moreover, we did not conduct primary culture tumor cells and further investigate on the chemoresistance profile of the tumor in vivo and in vitro.

In conclusion, the precise diagnosis of SS, especially poorly differentiated SS at an unusual site, is often a challenge to pathologists and clinical oncologists. Existing antibody panels, when correctly selected, have a major role in narrowing the differential diagnosis of soft tissue sarcomas. The SS18::SSX IHC with the extremely high specificity is a potential alternative that can be used to confirm the diagnosis of synovial sarcoma. Bone and soft tissue tumors, especially small round-cell malignancies, are sometimes difficult to be diagnosed by routine pathological examination such as IHC or FISH, and the advent of NGS has been helpful to identify the presence of molecular genetic abnormalities that can be applied to the final diagnosis.

## Materials and Methods

### Immunohistochemistry

Cytomorphological features of tumor tissues were analyzed through hematoxylin and eosin (H&E) staining. Briefly, resected tumor tissue was paraffin embedded and sectioned into 5-µm-thick slices using a microtome and stained using standard techniques. Protein expression was assessed by immunohistochemical analysis as previously reported (62). In brief, 5-µm-thick sections cut from paraffin-embedded tumor tissues were de-paraffinized with xylene for 1 h, then rehydrated and incubated with antigen retrieval solution in a water bath at 98.5°C for 30 min. After cooling, the sections were incubated for 10 min with 3% hydrogen peroxide solution and washed twice with demineralized water. Next, they were incubated with a 3% bovine serum albumin solution in PBS for 20 min and then incubated with antibody (CD56, Bcl-2, TLE1, C99,CD57, CK, CK8/18, Calponin, Syn, Vim, FLI-1, NKX2.2, CgA, S100, NSE, LCA, Desmin, WT-1, SATB2, TTF-1, Myogenin, MyoD1, SMA, PD-1, PD-L1, and Ki-67) at room temperature for 1 h. Staining was revealed using the streptavidin-biotinperoxidase complex (ABC) method. Cell nuclei were counterstained with hematoxylin (Sigma Aldrich, Saint Louis, MO, USA). Cells were considered positive in the presence of brown nuclear immunostaining.

### Fluorescence *In Situ* Hybridization

FISH was performed and evaluated according to previously published methods. Briefly, 4 μm sections of formalin-fixed paraffin-embedded (FFPE) tissue were hybridized with fluorescent probes LSI SS18 mapped to 18q11.2 (Vysis, Guangzhou Anbiping [LBP] Pharmaceutical Technology Co., Ltd., China) and counterstained with 4,6-diamidino-2-phenylindole. Two hundred consecutive nuclei showing complete (i.e., two green and two orange) signals were scored with the threshold of 20% break-apart signals set as a positive result. Nuclei with incomplete signals were omitted.

### Sample Collection and Sequencing

All of the FFPE samples were transferred to the OrigiMed (Shanghai, China) for a NGS analysis with a YuanSuTM panel 1 at a CAP-accredited and CLIA-certified laboratory. Informed consent was obtained from all patients.

## Statistical Analysis

Genomic alterations including single-base substitutions, short and long insertions/deletions (Indels), copy number variations (CNVs), gene rearrangement, microsatellite instability (MSI), and tumor mutational burden (TMB) were assessed using the OrigiMed pipeline. The qualitative variables were analyzed by Fisher’s exact test. The comparisons of normal quantitative distributed data were performed using the *t*-test, and Wilcoxon rank test was used for the nonnormal distributed data. All of the statistical analyses were performed with SPSS 22.0.

## Data Availability Statement

The original contributions presented in the study are included in the article/**Supplementary Material**. Further inquiries can be directed to the corresponding authors.

## Ethics Statement

The patients/participants provided their written informed consent to participate in this study. Informed consent was obtained from the relevant individuals, and next of kin, for the publication of any potentially identifiable images or data included in this article.

## Author Contributions

KP and XG: their contribution was to design the project, collect the patients’ clinical history, review the literature, and write the paper. YJ and LX: their contribution was to characterize histological samples. LL and ZL: they directed the project and also helped to design and write the paper. All authors contributed to the article and approved the submitted version.

## Funding

This work was financially supported by the National Natural Science Foundation of China (No. 81902745), Hunan Provincial Research and Development Program in Key Areas (2019WK2071, 2020DK2003), and China Postdoctoral Science Foundation (No. 2021M693557).

## Conflict of Interest

The authors declare that the research was conducted in the absence of any commercial or financial relationships that could be construed as a potential conflict of interest.

## Publisher’s Note

All claims expressed in this article are solely those of the authors and do not necessarily represent those of their affiliated organizations, or those of the publisher, the editors and the reviewers. Any product that may be evaluated in this article, or claim that may be made by its manufacturer, is not guaranteed or endorsed by the publisher.
