# Supplementary figures and images for: Case Report: Primary Intraosseous Poorly Differentiated Synovial Sarcoma of the Femur
Source: Front Oncol. 2022 Mar 16;12:754131. doi: 10.3389/fonc.2022.754131 (PMC8966429; doi:10.3389/fonc.2022.754131)

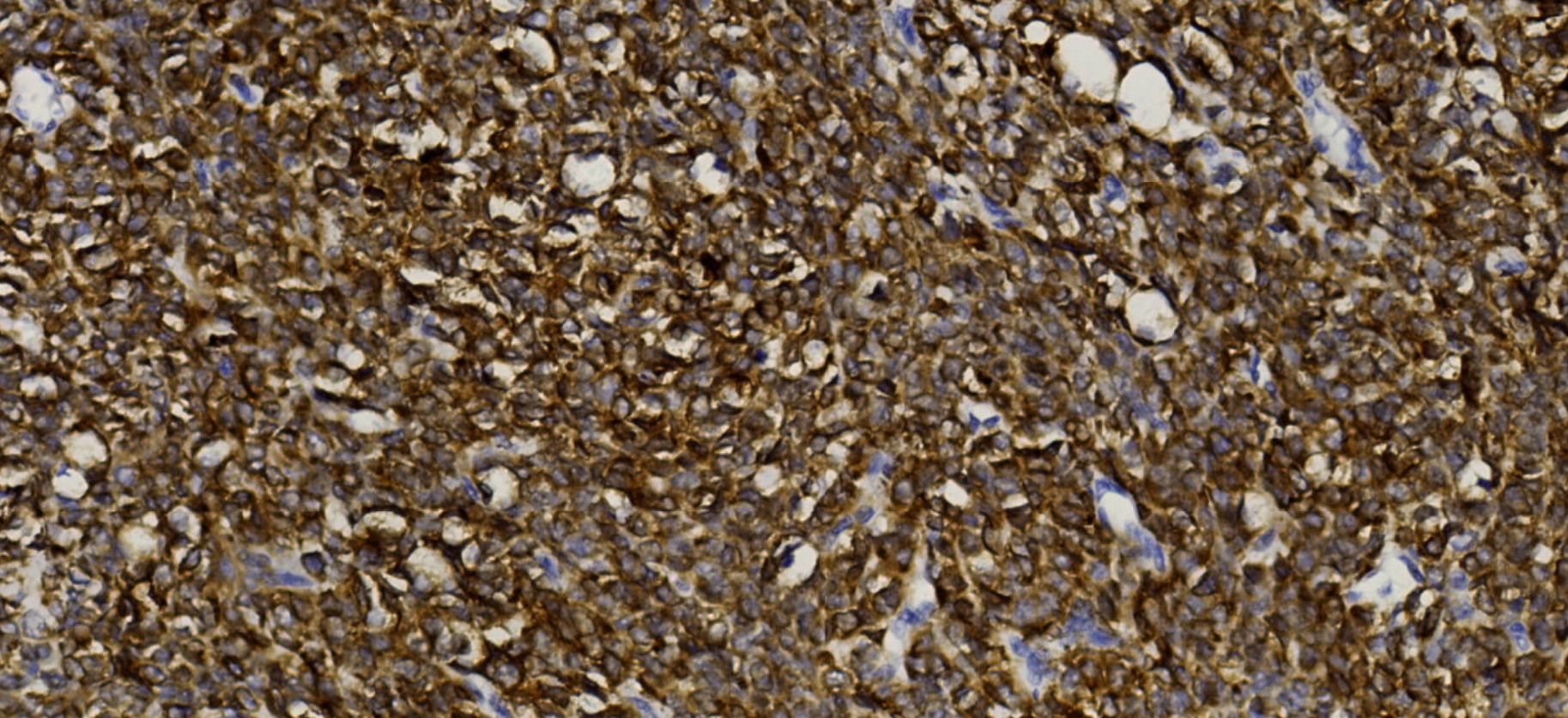

Supplement: Supplementary file 1 [file DataSheet_1.zip › Supplementary Materials/Bcl-2 Surgical specimens.jpg]

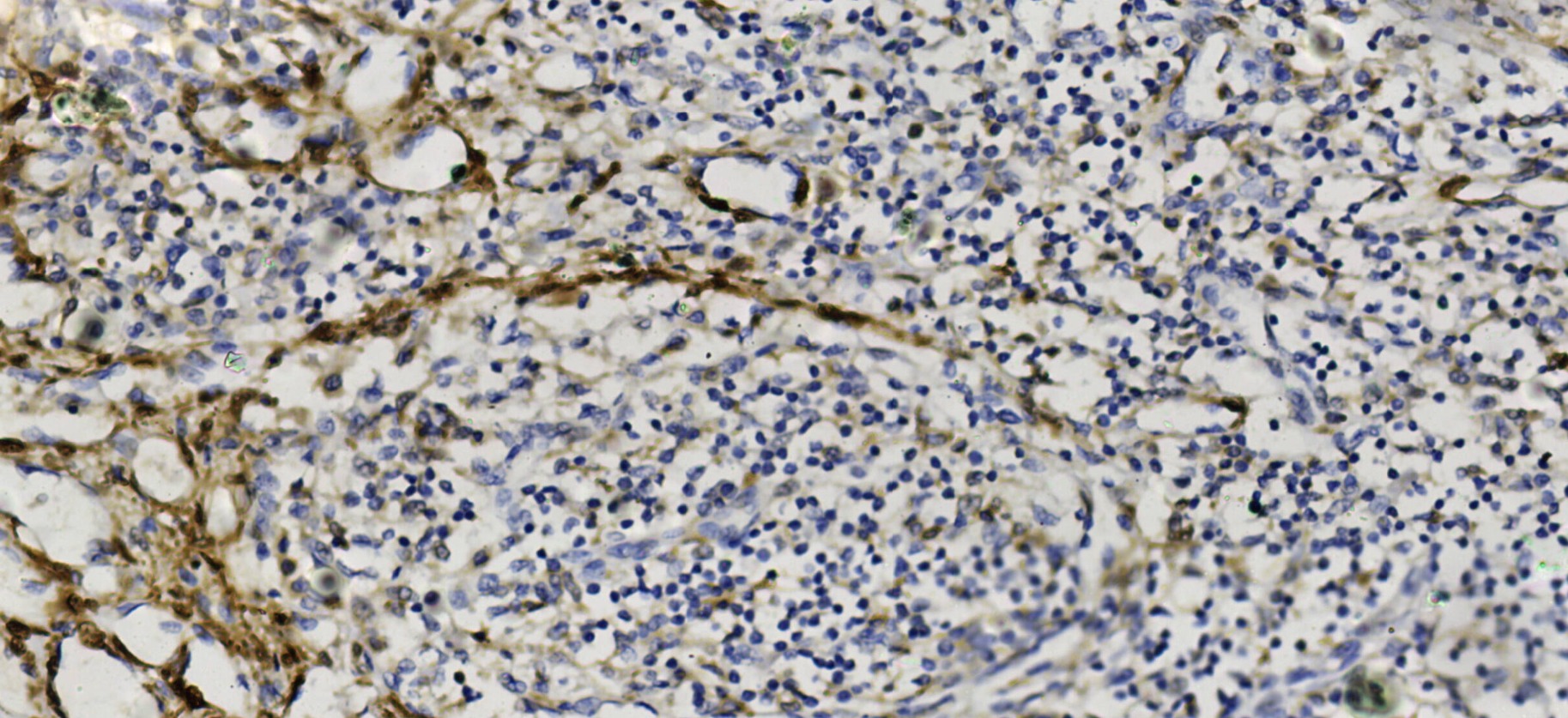

Supplement: Supplementary file 1 [file DataSheet_1.zip › Supplementary Materials/Calponin-Surgical specimens.jpg]

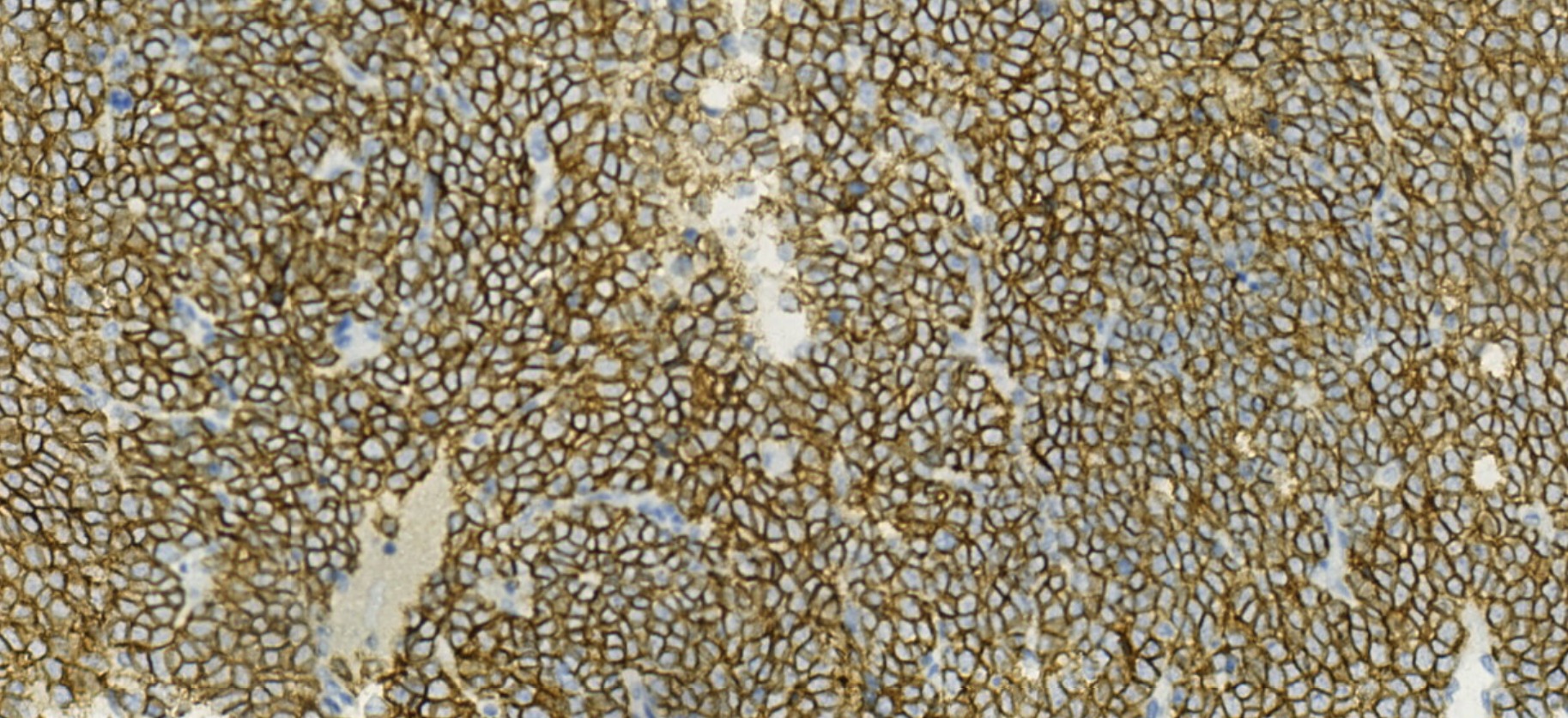

Supplement: Supplementary file 1 [file DataSheet_1.zip › Supplementary Materials/CD56 biopsy specimen.jpg]

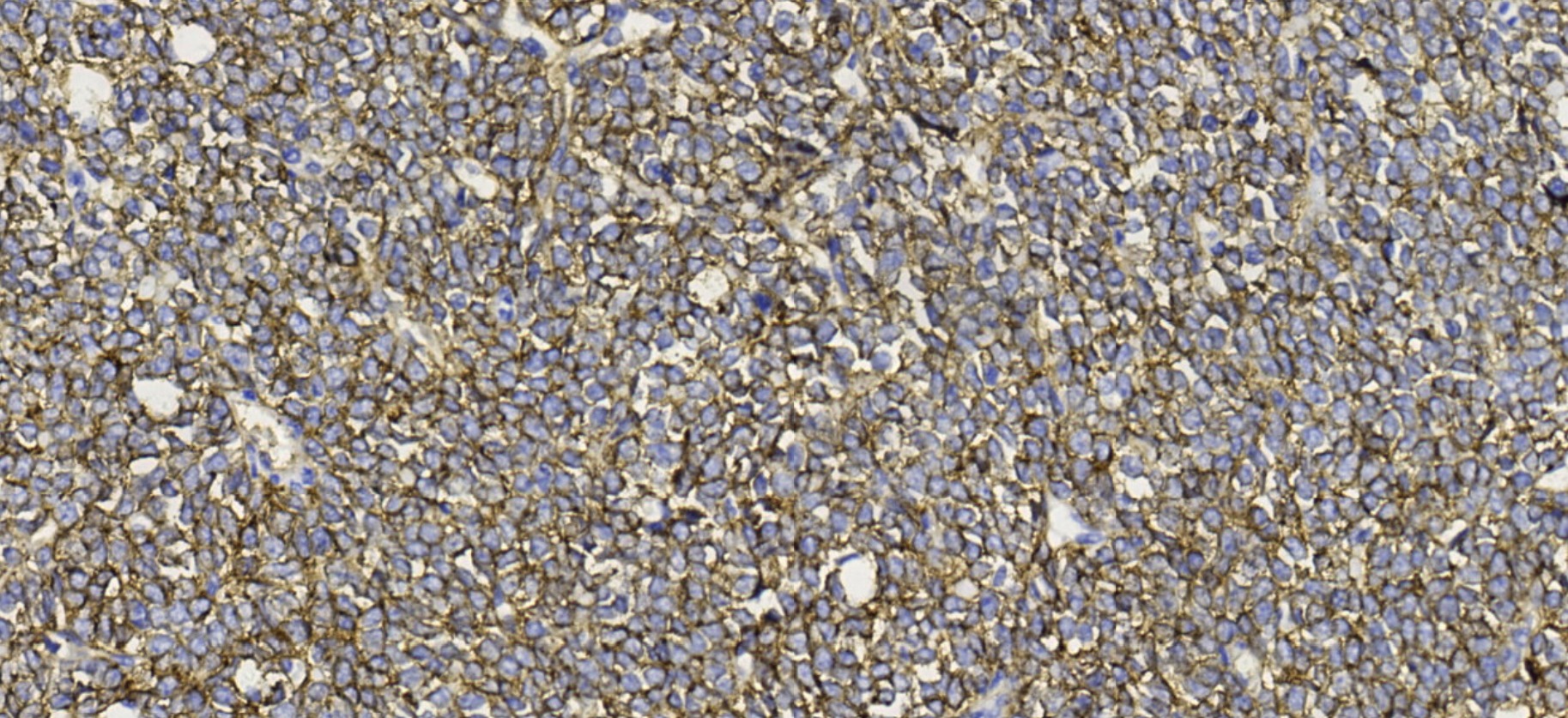

Supplement: Supplementary file 1 [file DataSheet_1.zip › Supplementary Materials/CD56 Surgical specimens.jpg]

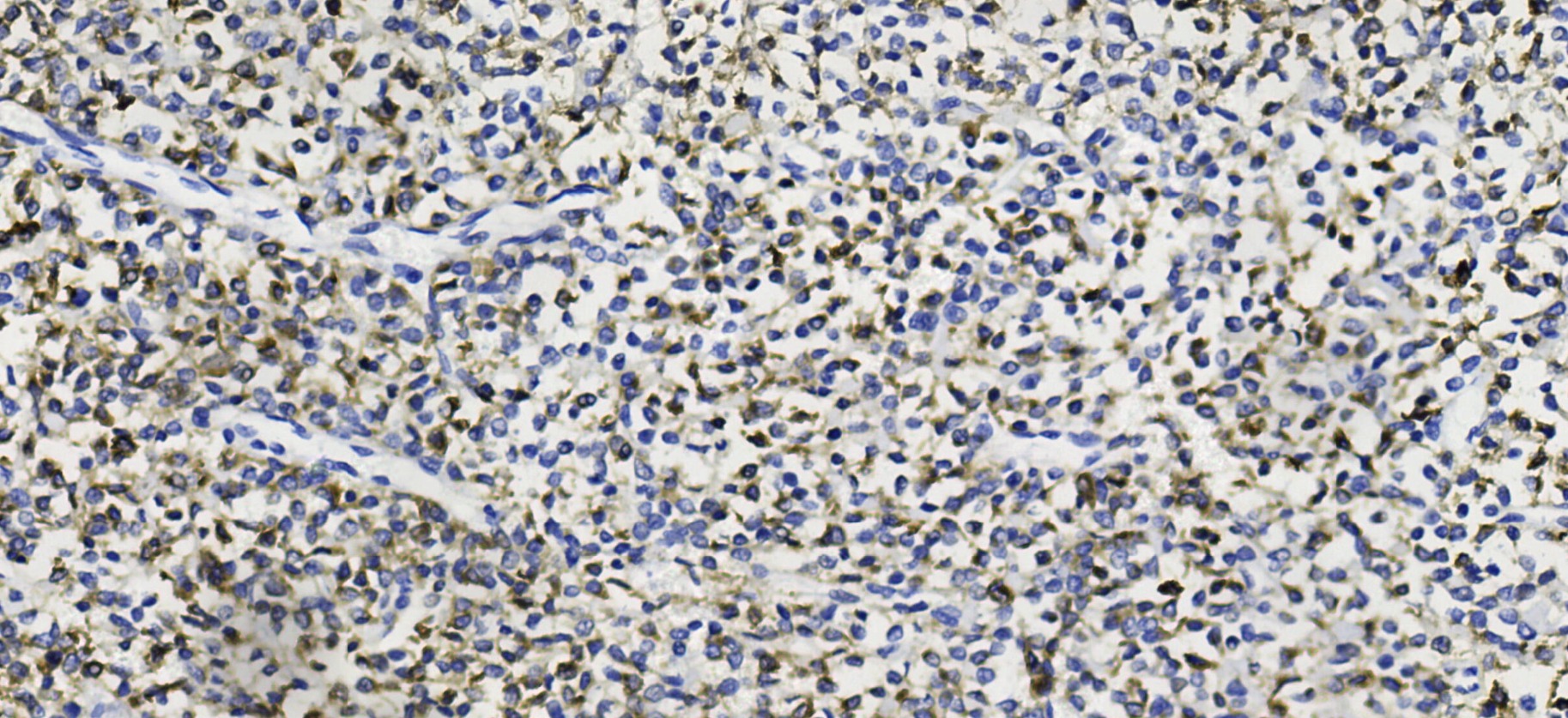

Supplement: Supplementary file 1 [file DataSheet_1.zip › Supplementary Materials/CD57-Surgical specimens.jpg]

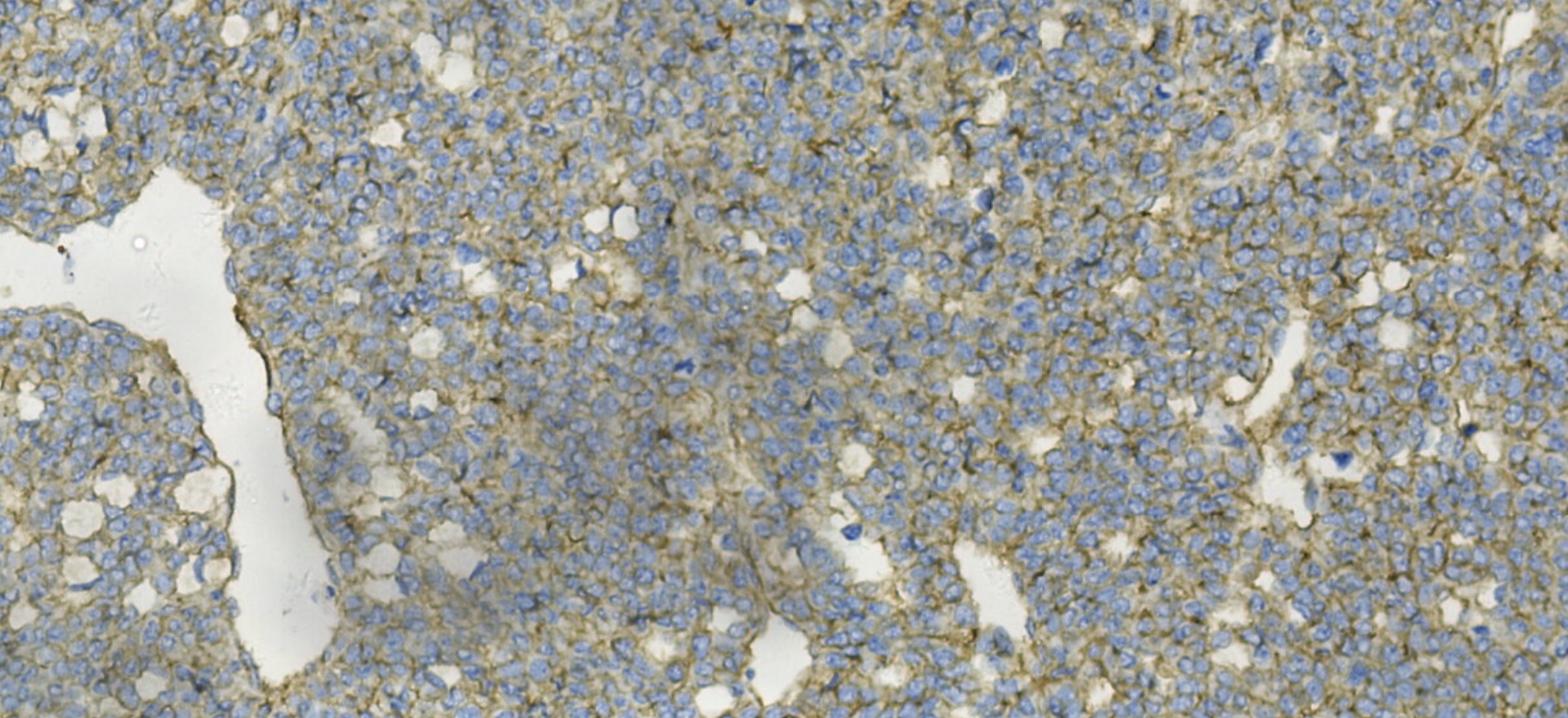

Supplement: Supplementary file 1 [file DataSheet_1.zip › Supplementary Materials/CD99 biopsy specimen.jpg]

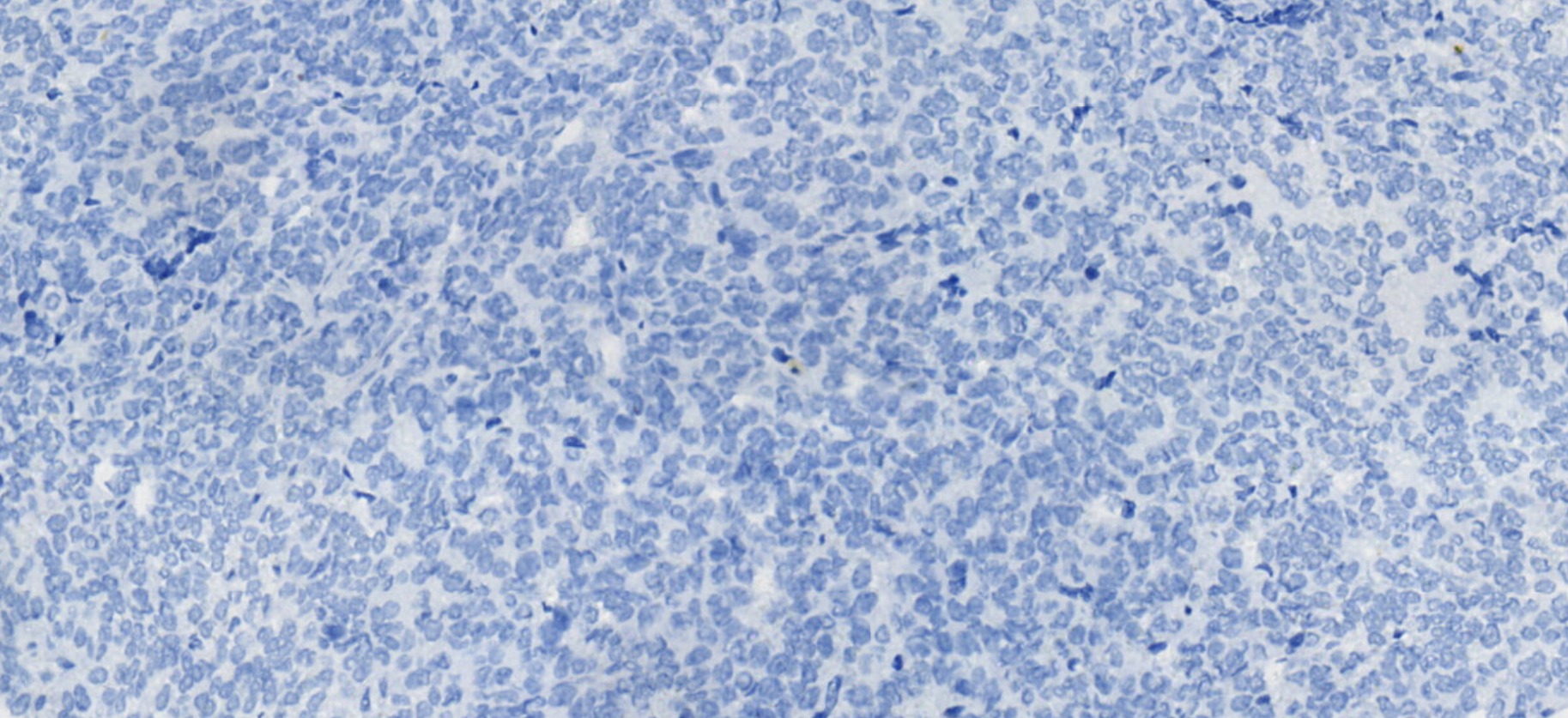

Supplement: Supplementary file 1 [file DataSheet_1.zip › Supplementary Materials/CgA biopsy specimen.jpg]

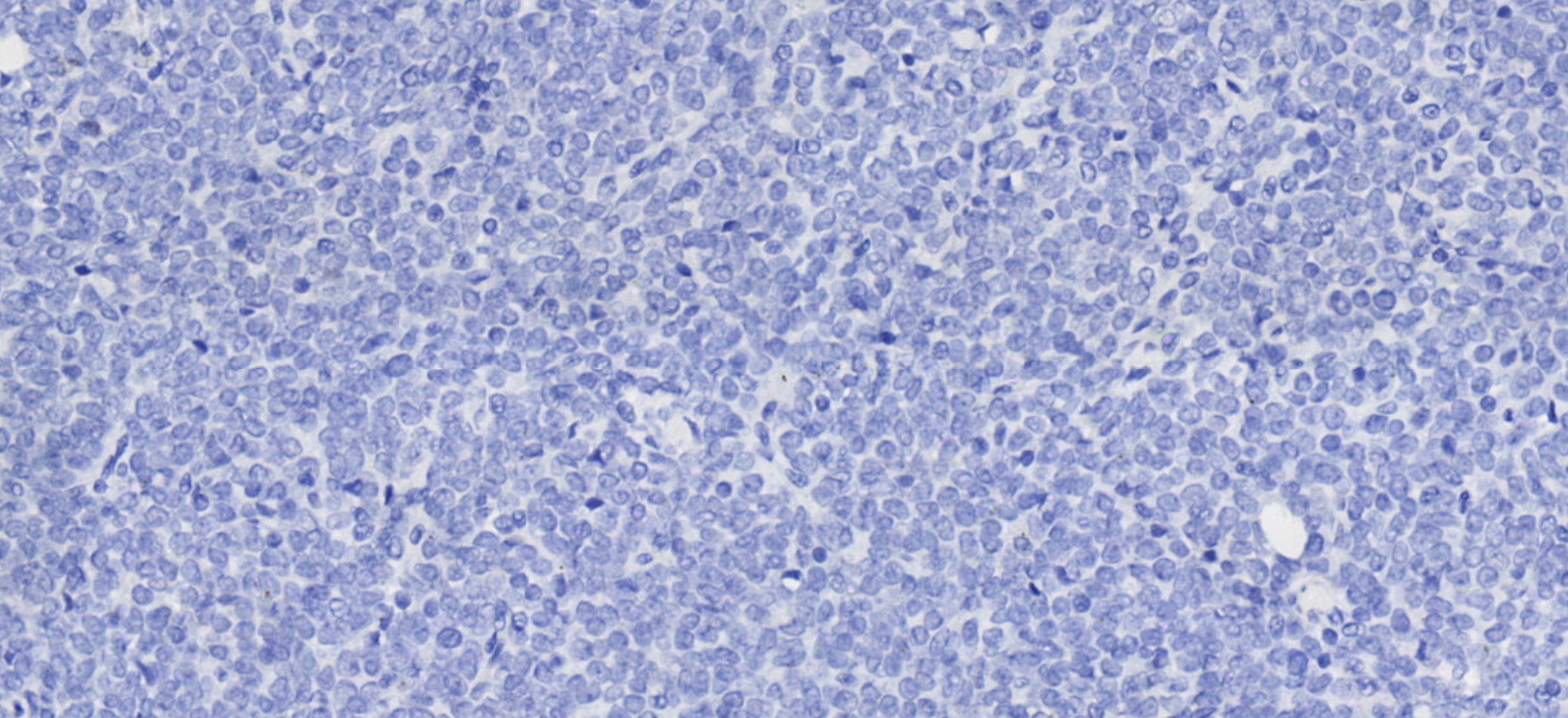

Supplement: Supplementary file 1 [file DataSheet_1.zip › Supplementary Materials/CgA Surgical specimens.jpg]

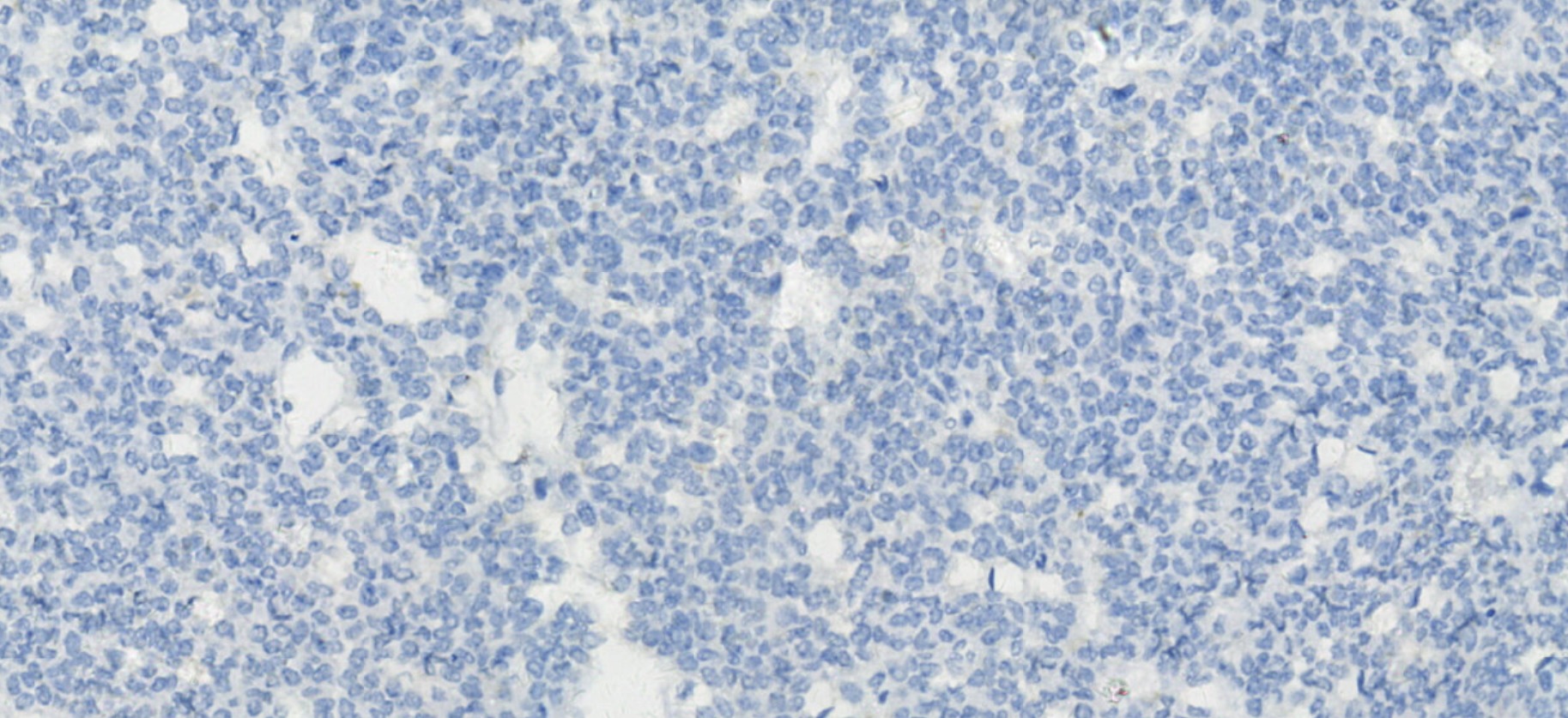

Supplement: Supplementary file 1 [file DataSheet_1.zip › Supplementary Materials/CK biopsy specimen.jpg]

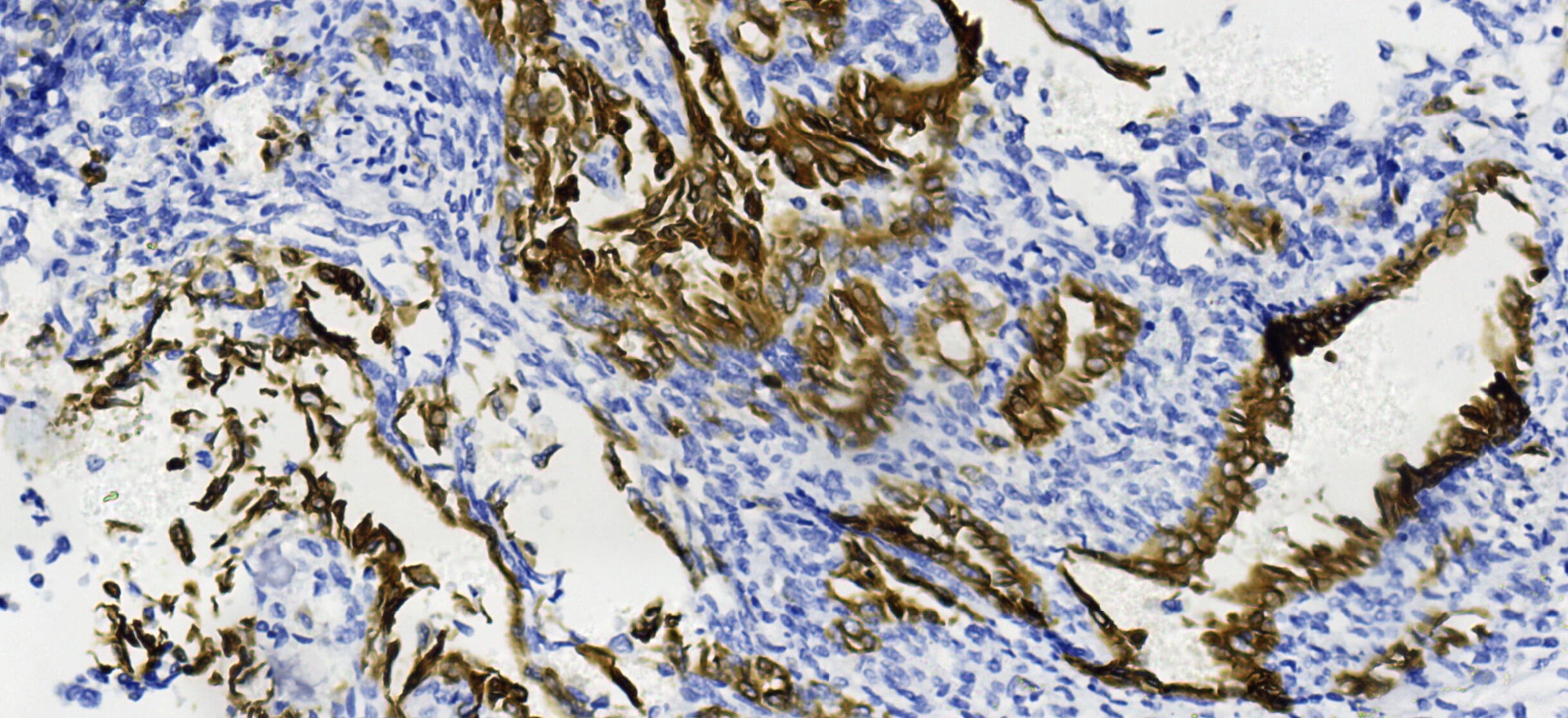

Supplement: Supplementary file 1 [file DataSheet_1.zip › Supplementary Materials/CK-Surgical specimens.jpg]

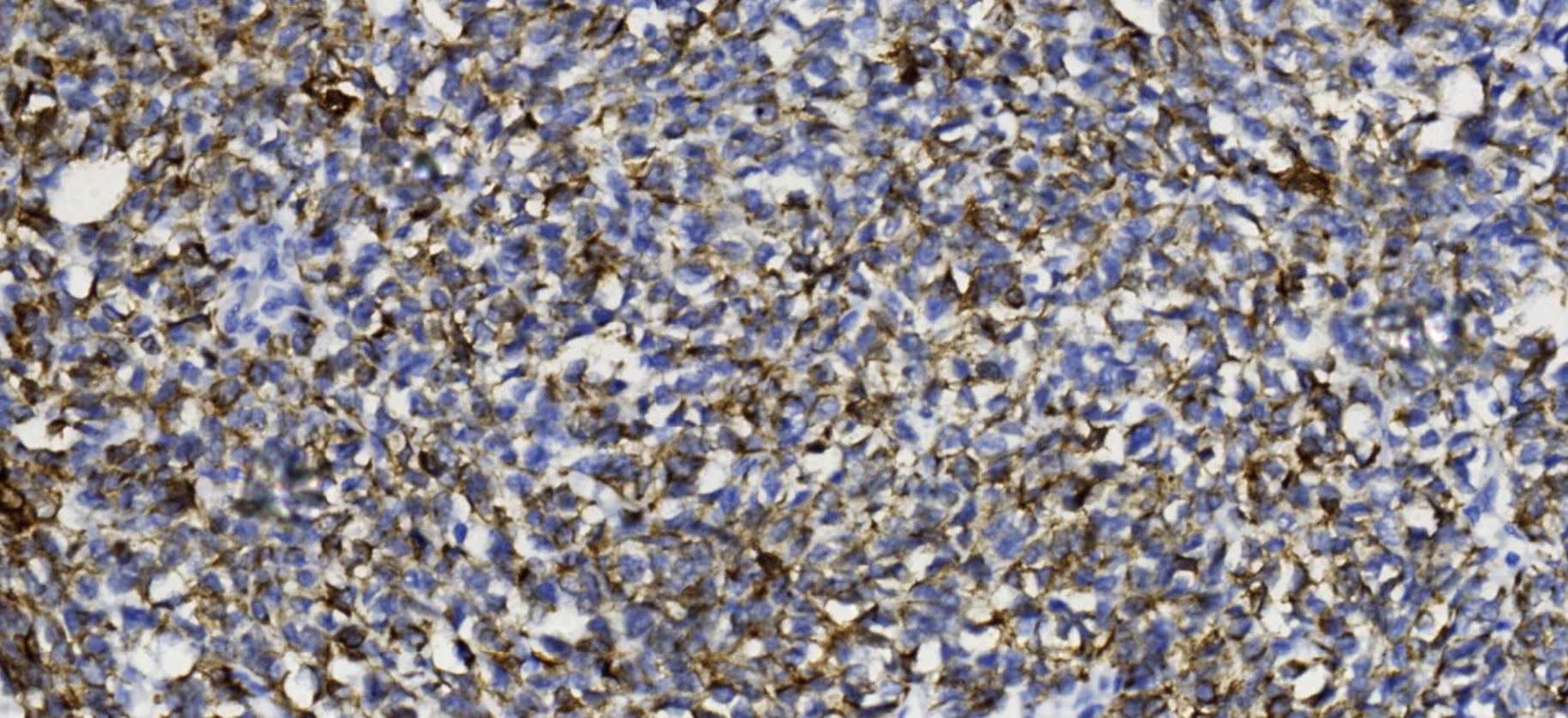

Supplement: Supplementary file 1 [file DataSheet_1.zip › Supplementary Materials/CK8 18-Surgical specimens.jpg]

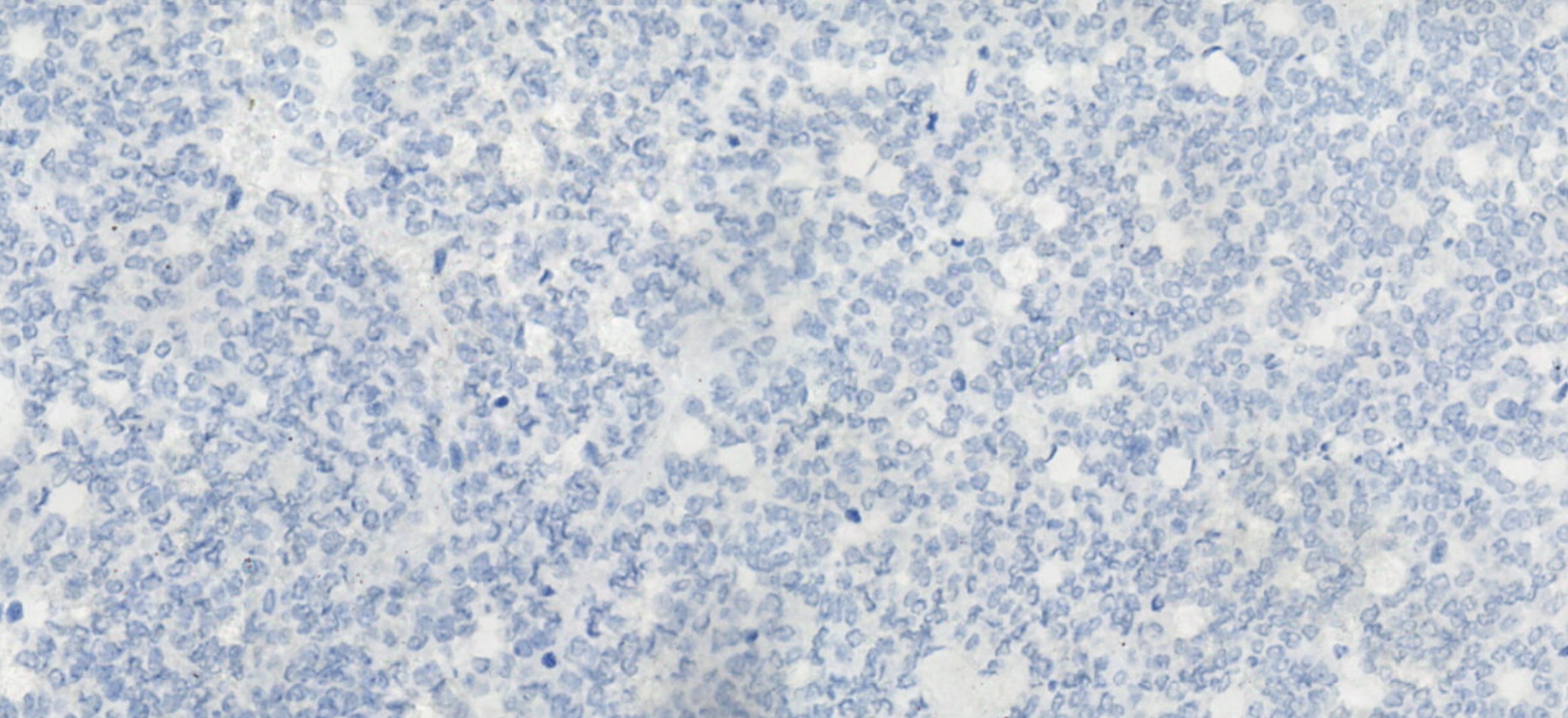

Supplement: Supplementary file 1 [file DataSheet_1.zip › Supplementary Materials/Desmin biopsy specimen.jpg]

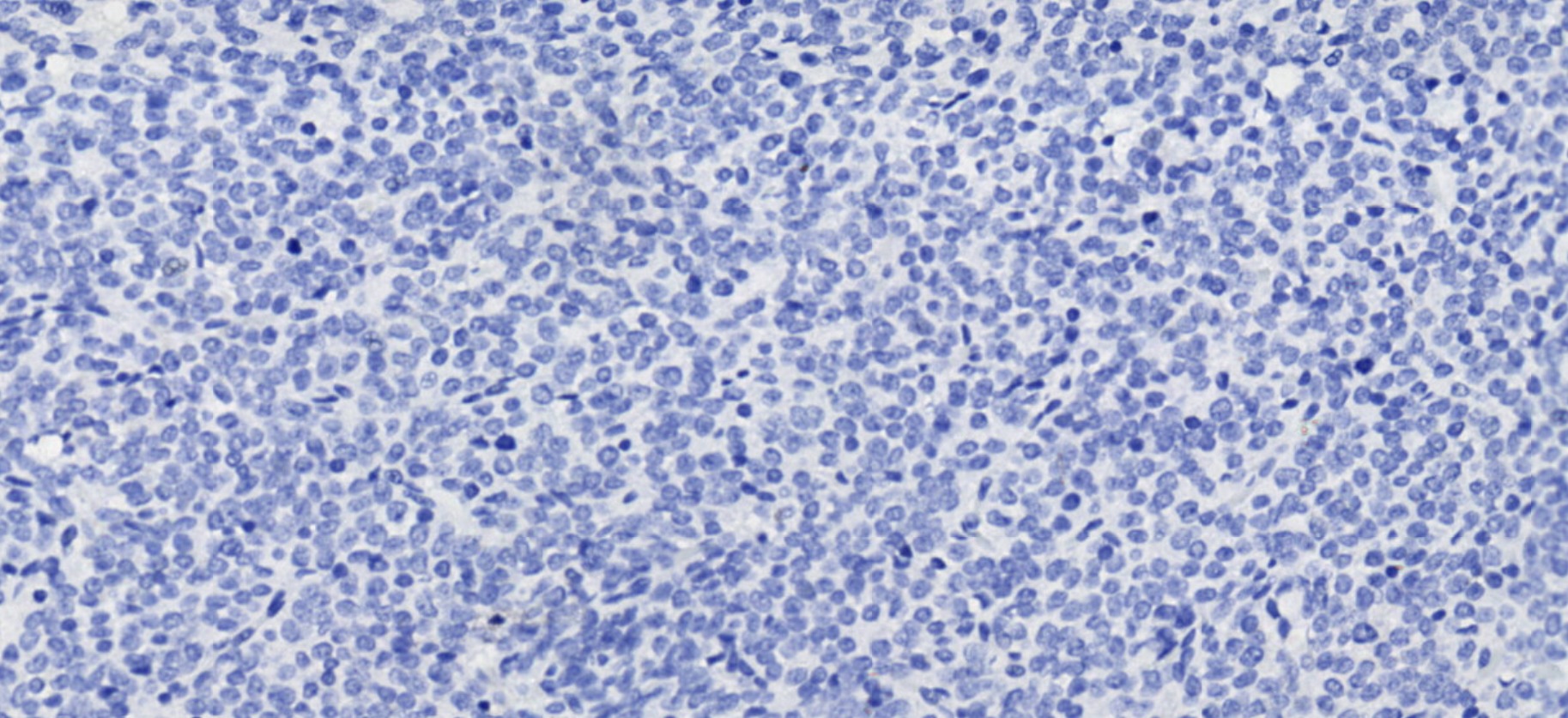

Supplement: Supplementary file 1 [file DataSheet_1.zip › Supplementary Materials/Desmin Surgical specimens.jpg]

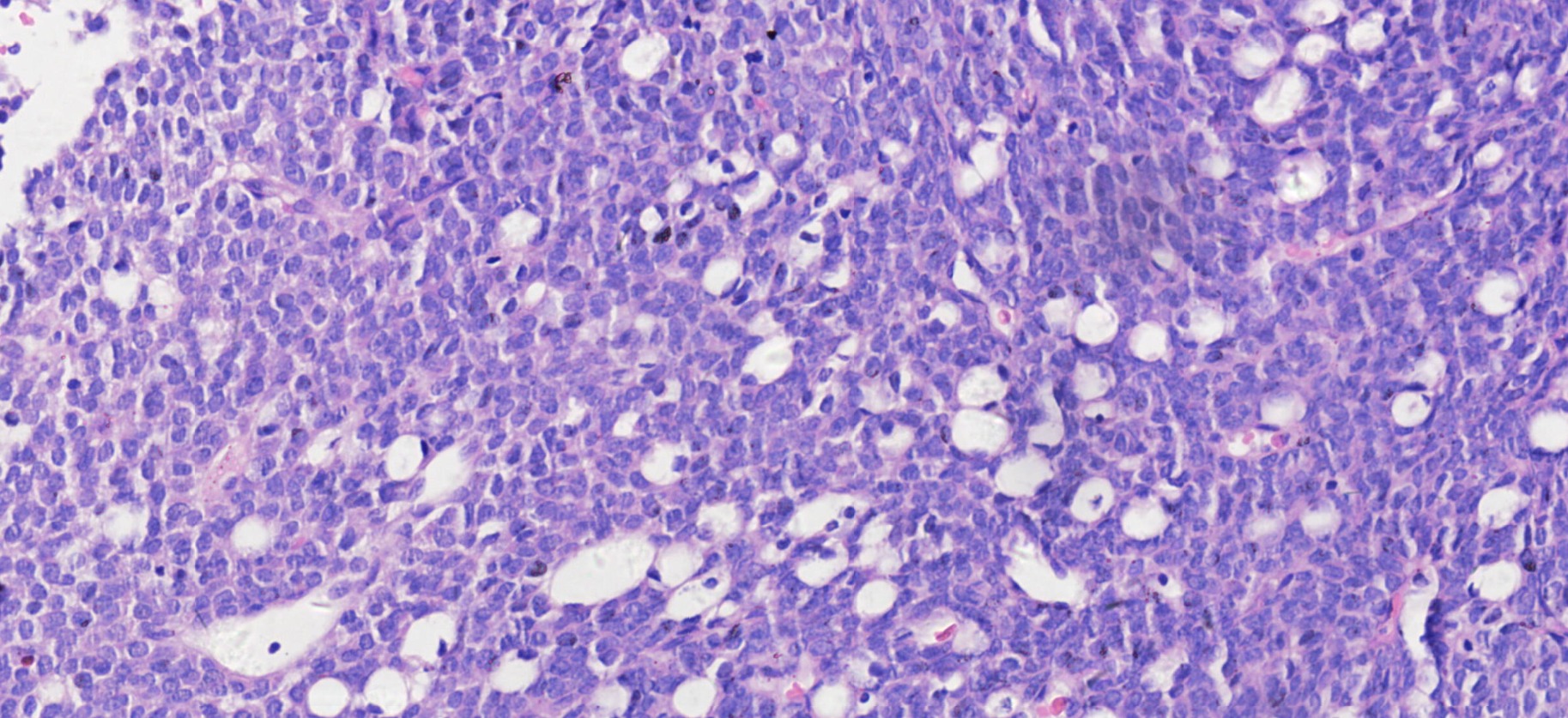

Supplement: Supplementary file 1 [file DataSheet_1.zip › Supplementary Materials/HE biopsy specimen.jpg]

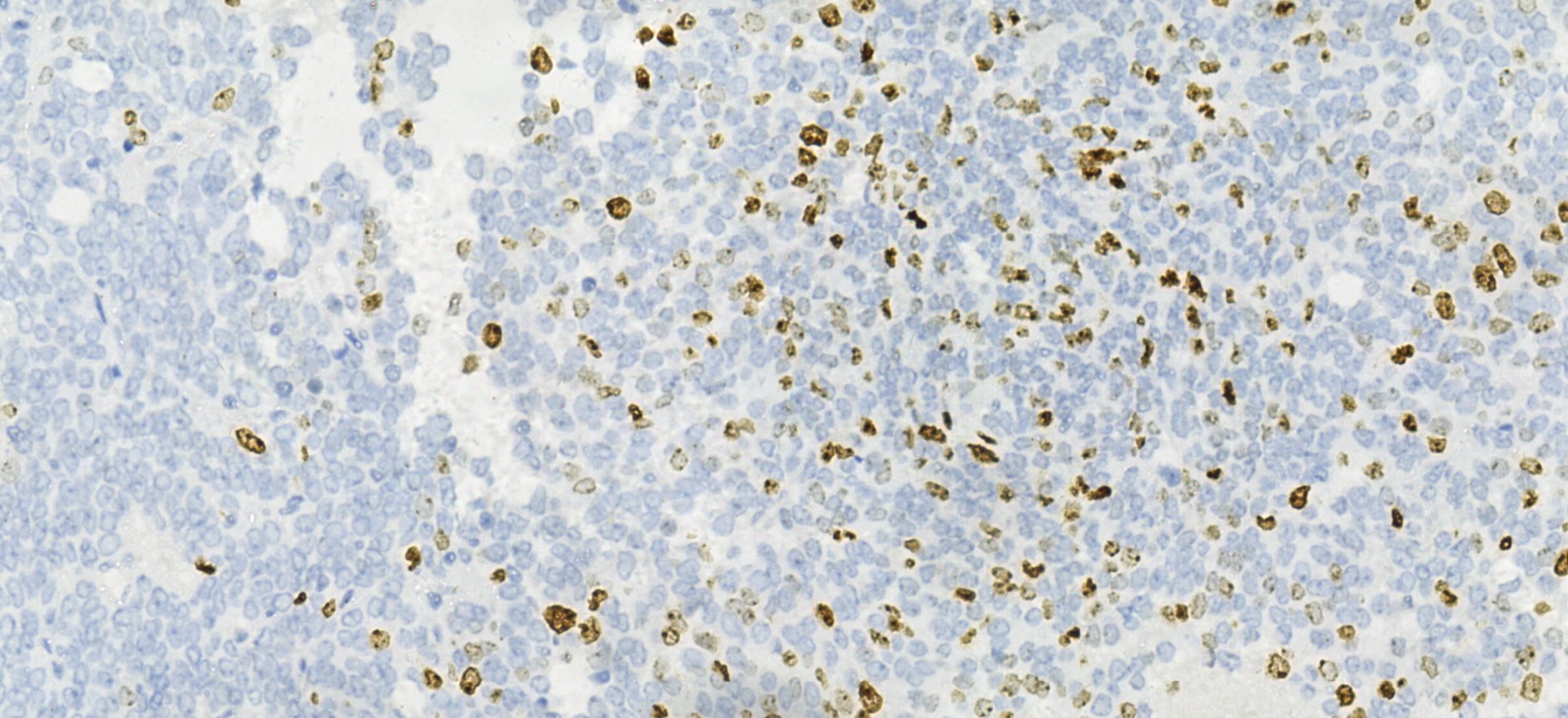

Supplement: Supplementary file 1 [file DataSheet_1.zip › Supplementary Materials/Ki67 biopsy specimen.jpg]

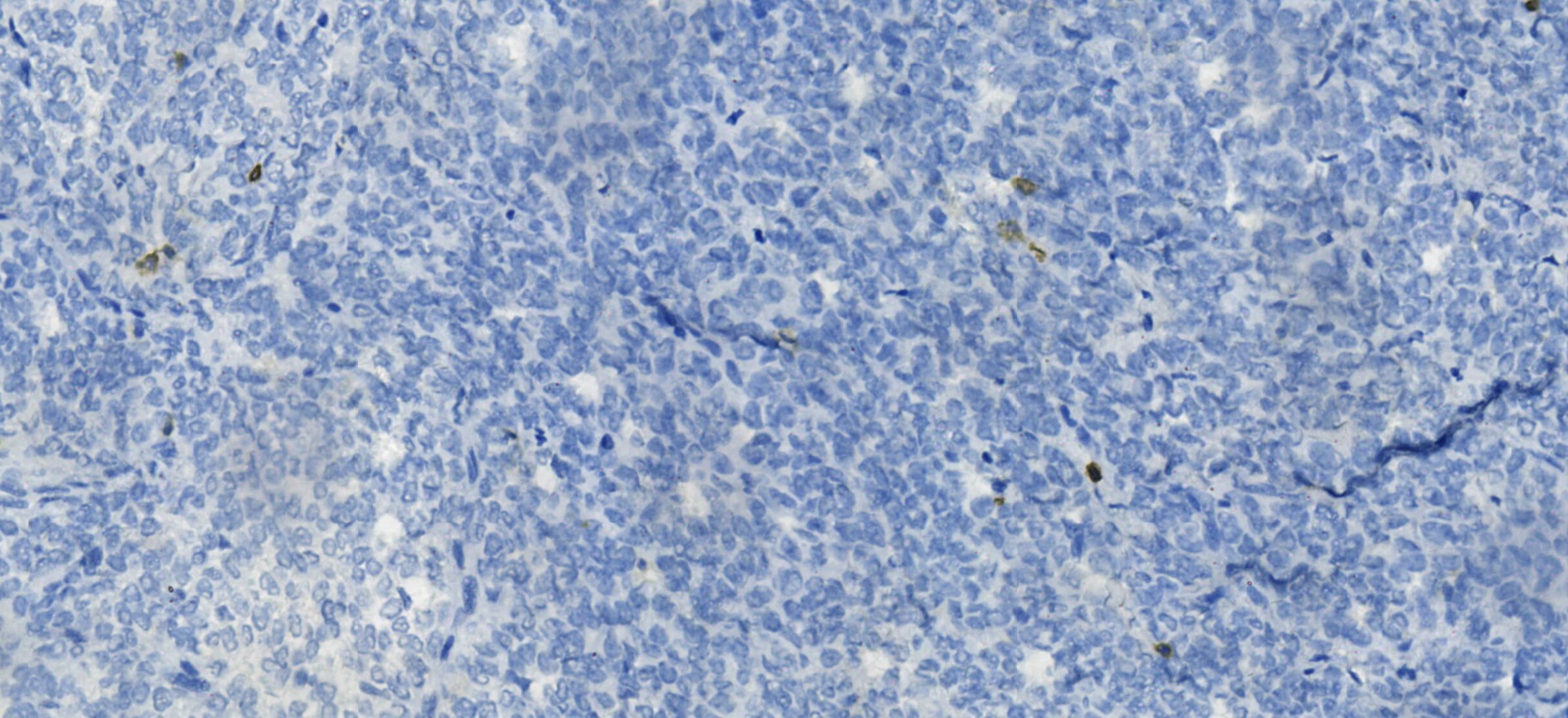

Supplement: Supplementary file 1 [file DataSheet_1.zip › Supplementary Materials/LCA biopsy specimen.jpg]

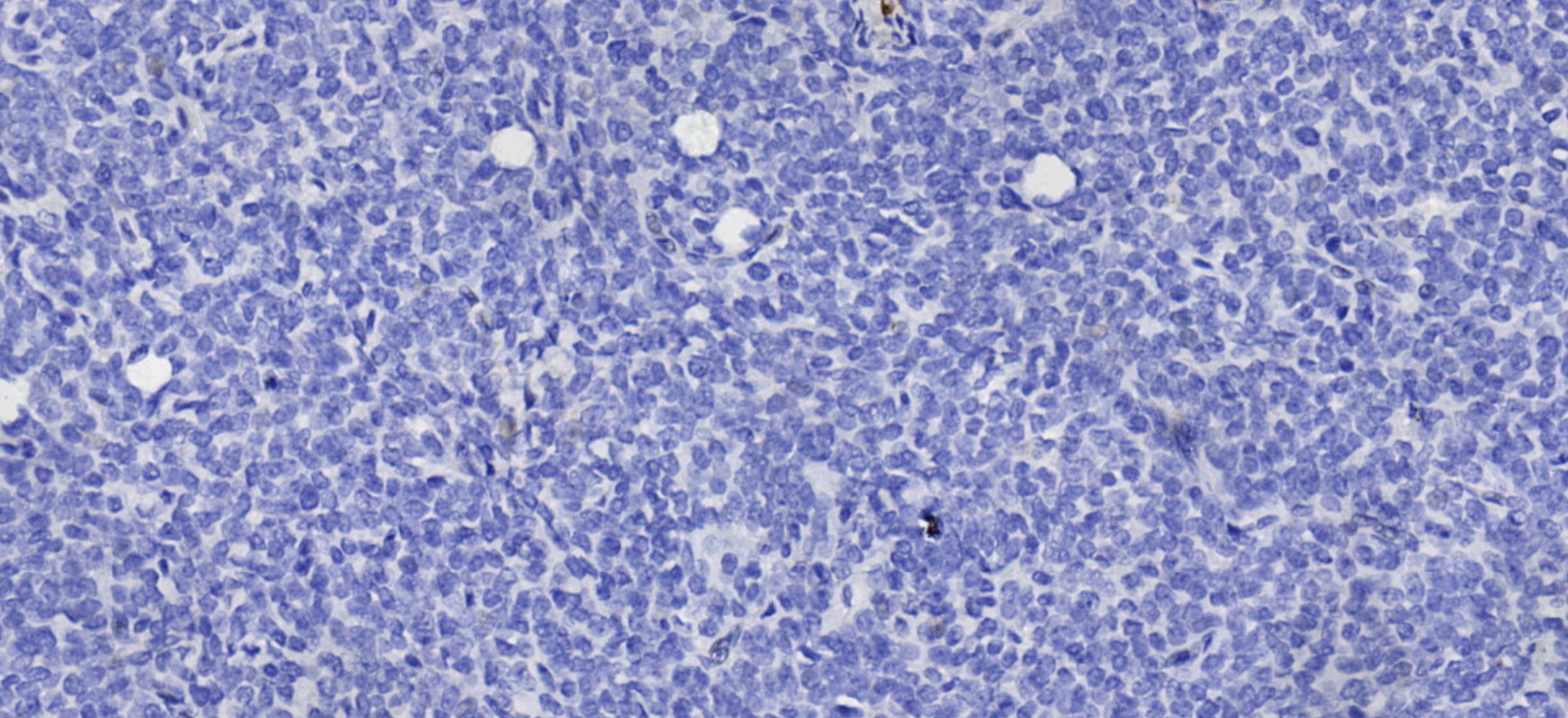

Supplement: Supplementary file 1 [file DataSheet_1.zip › Supplementary Materials/LCA Surgical specimens.jpg]

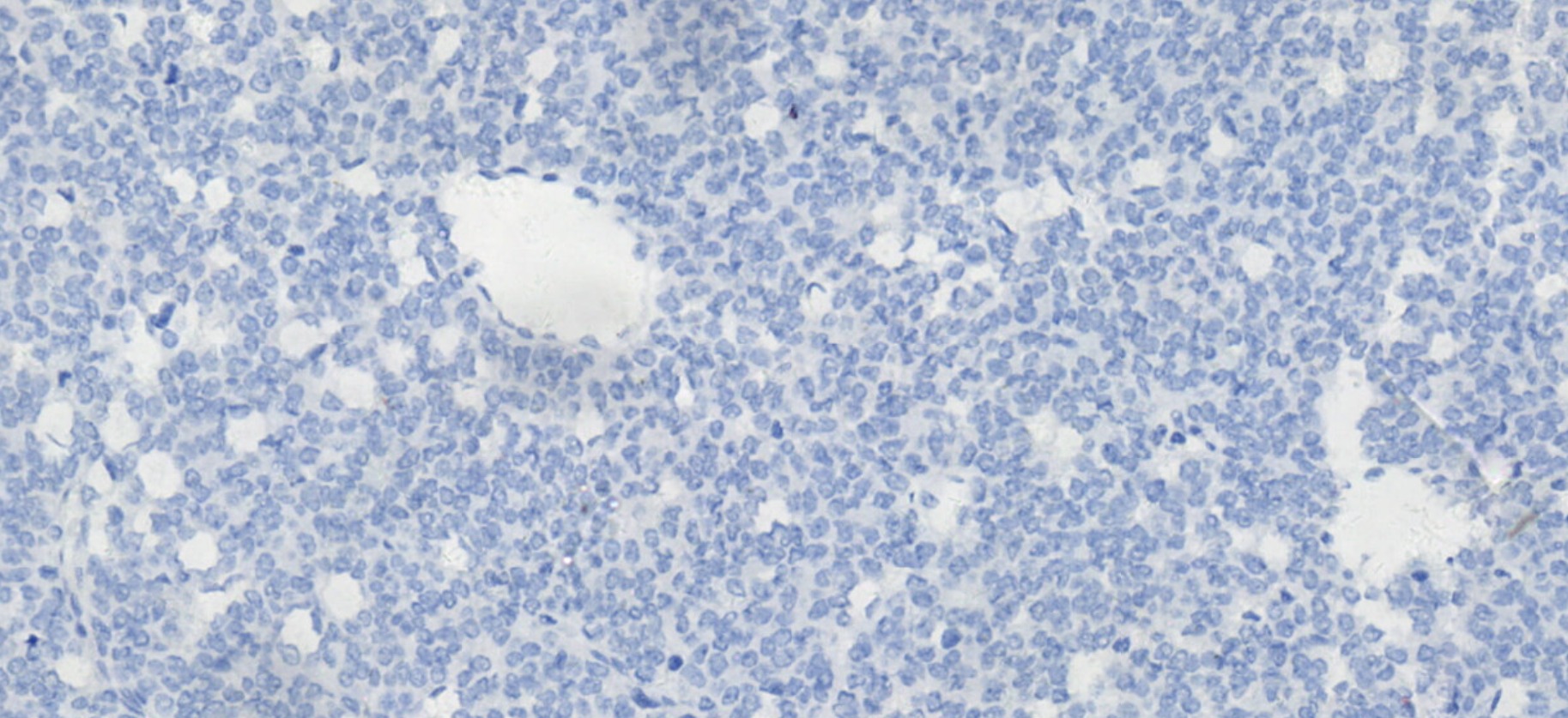

Supplement: Supplementary file 1 [file DataSheet_1.zip › Supplementary Materials/MyoD1 biopsy specimen.jpg]

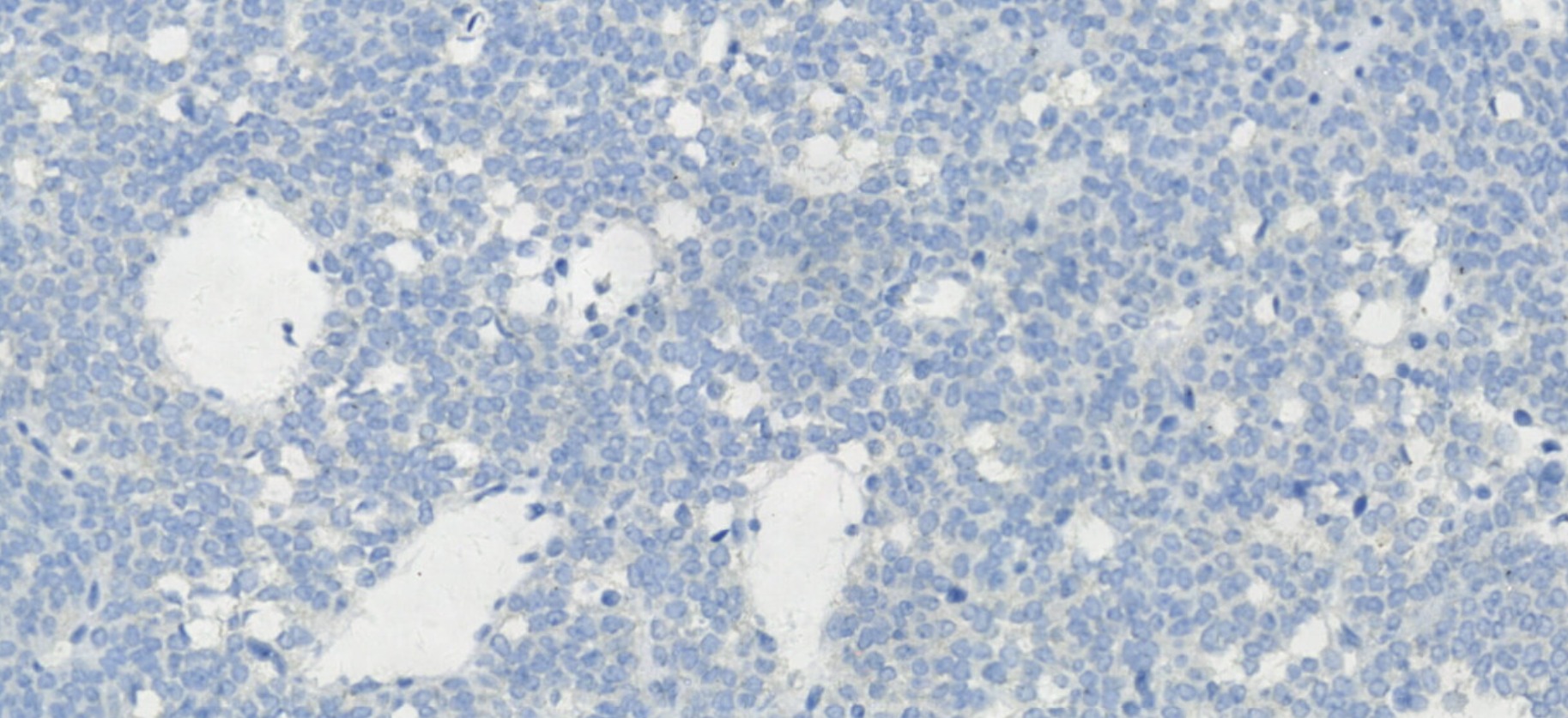

Supplement: Supplementary file 1 [file DataSheet_1.zip › Supplementary Materials/Myogenin biopsy specimen.jpg]

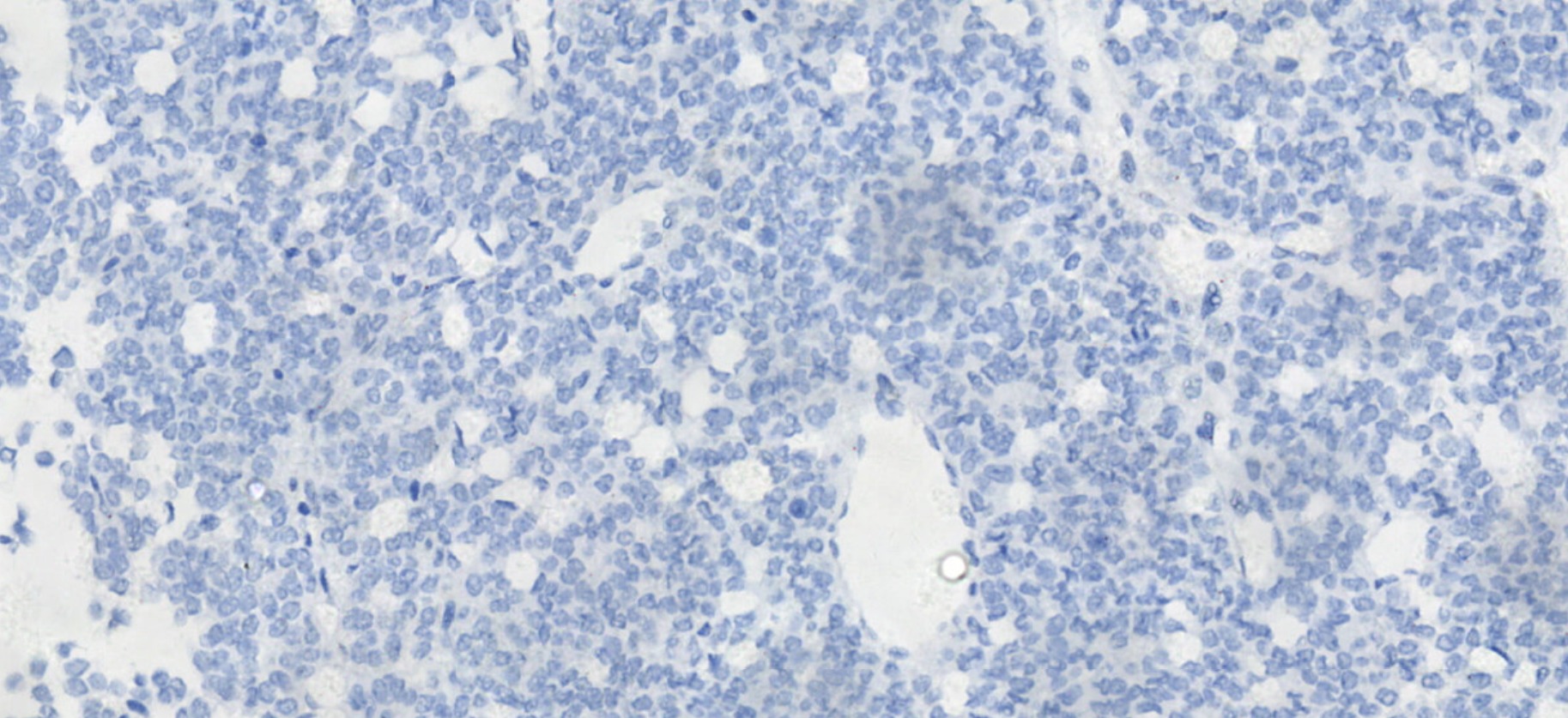

Supplement: Supplementary file 1 [file DataSheet_1.zip › Supplementary Materials/NKX2.2 biopsy specimen.jpg]

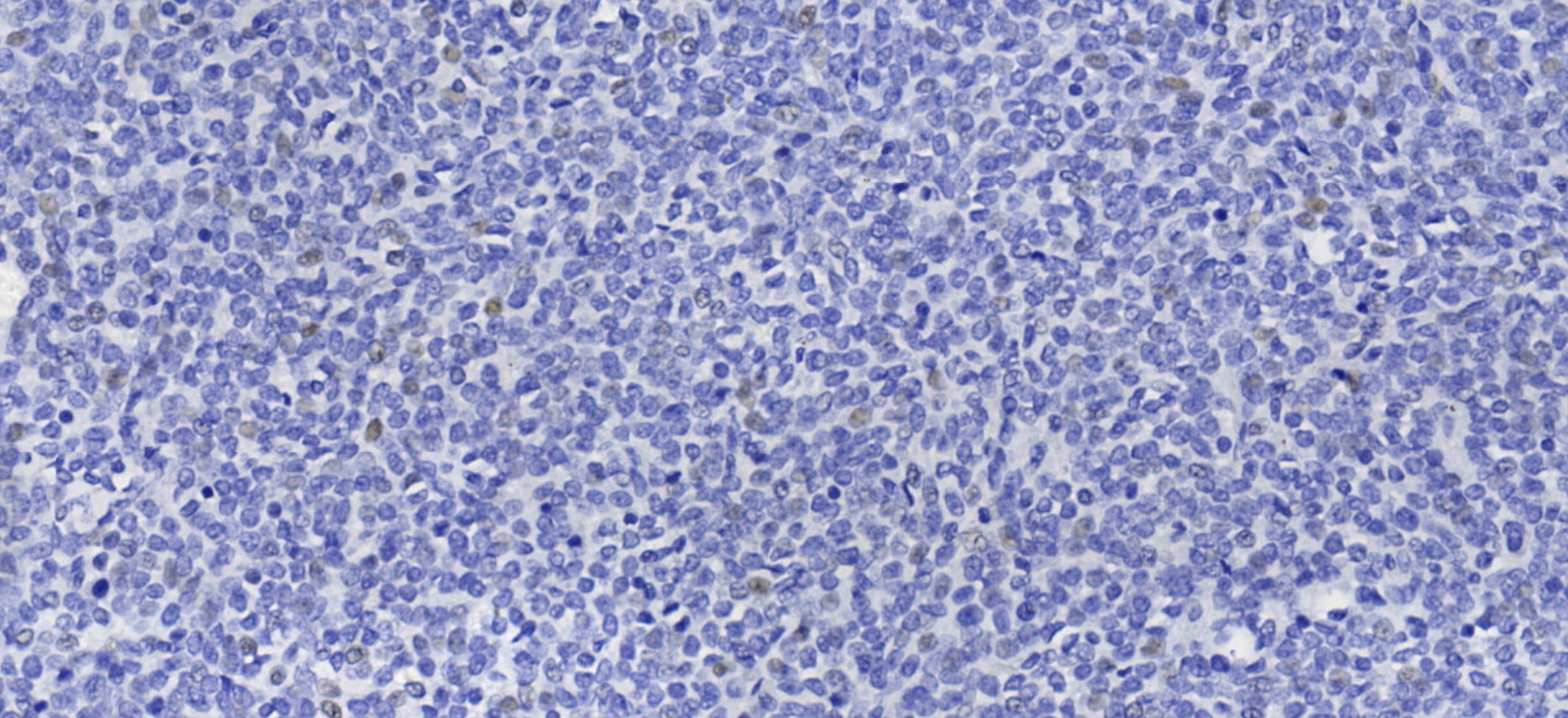

Supplement: Supplementary file 1 [file DataSheet_1.zip › Supplementary Materials/NKX2.2 Surgical specimens.jpg]

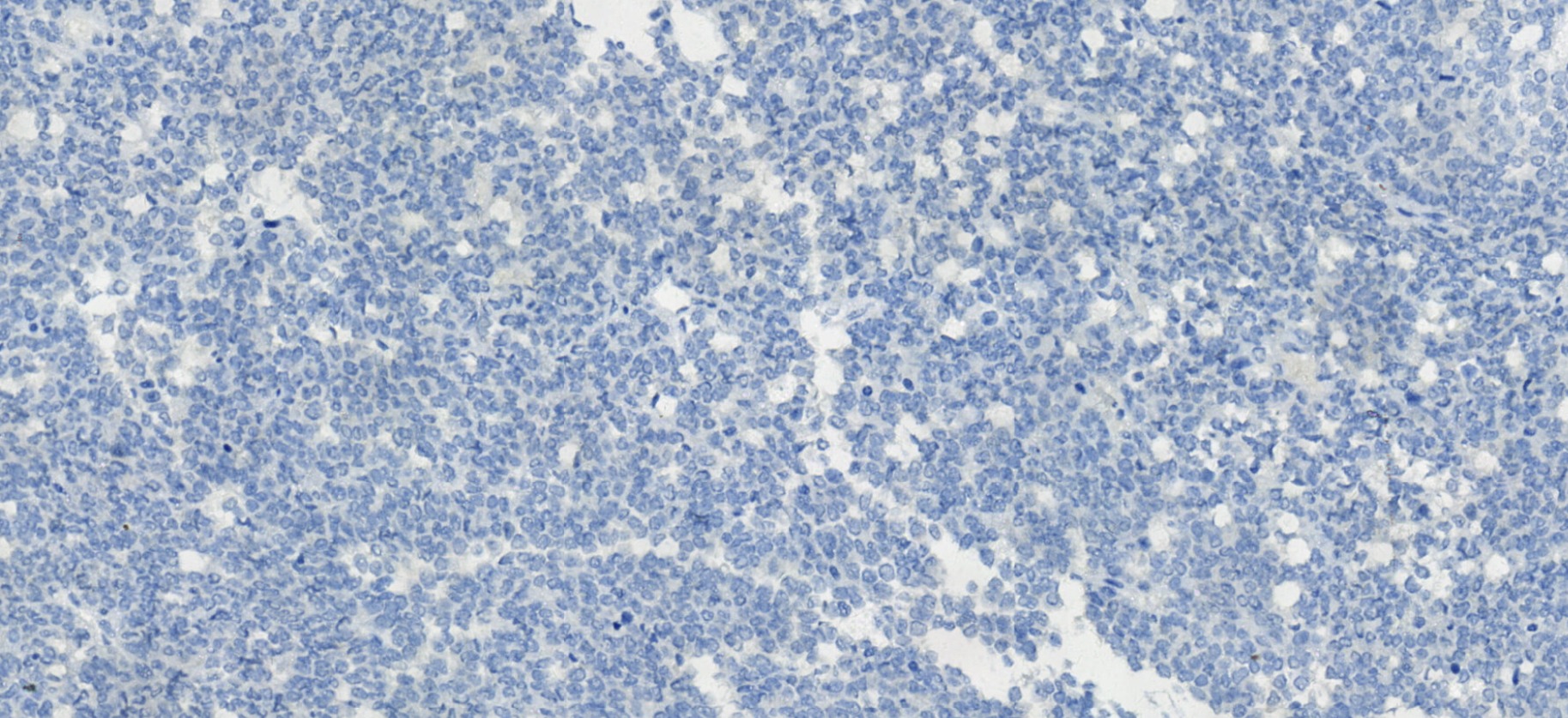

Supplement: Supplementary file 1 [file DataSheet_1.zip › Supplementary Materials/NSE biopsy specimen.jpg]

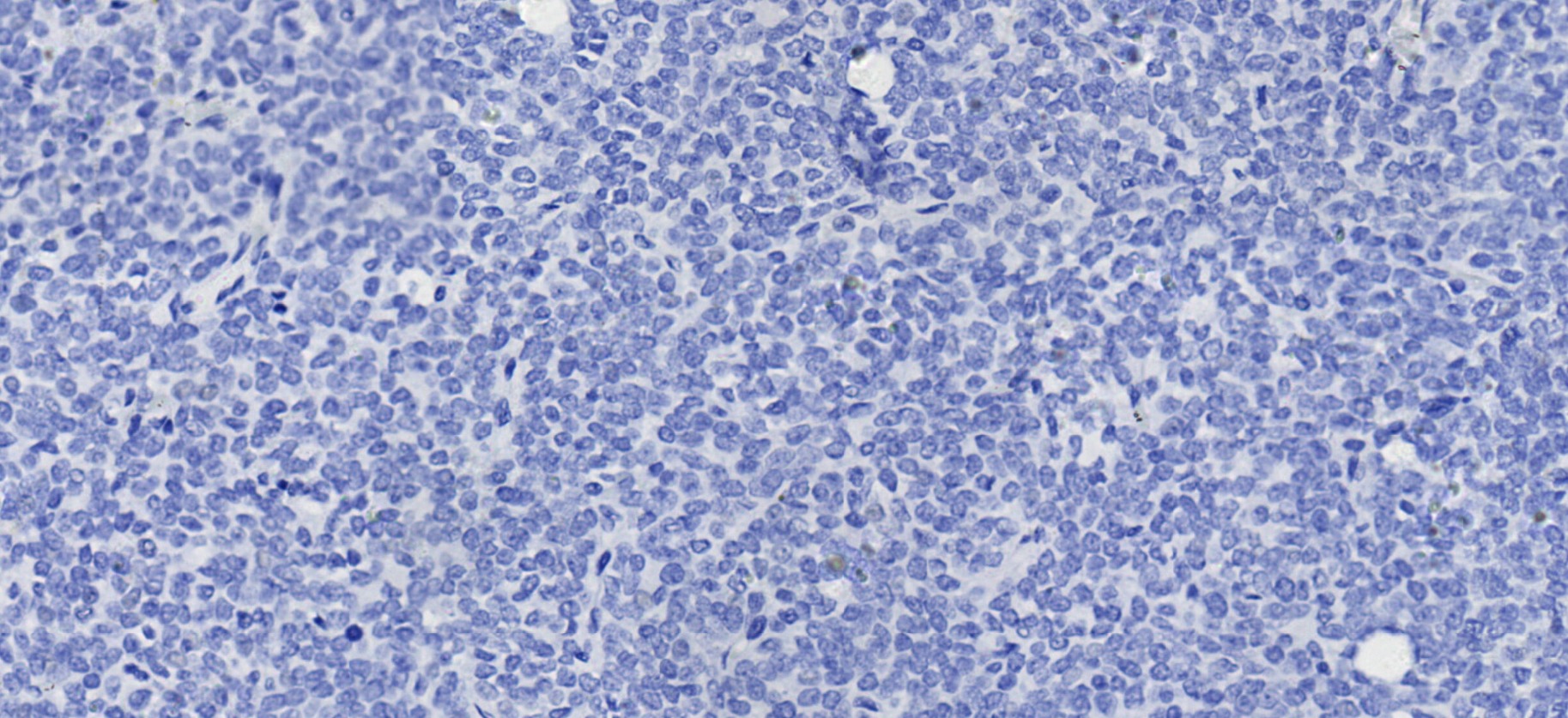

Supplement: Supplementary file 1 [file DataSheet_1.zip › Supplementary Materials/NSE Surgical specimens.jpg]

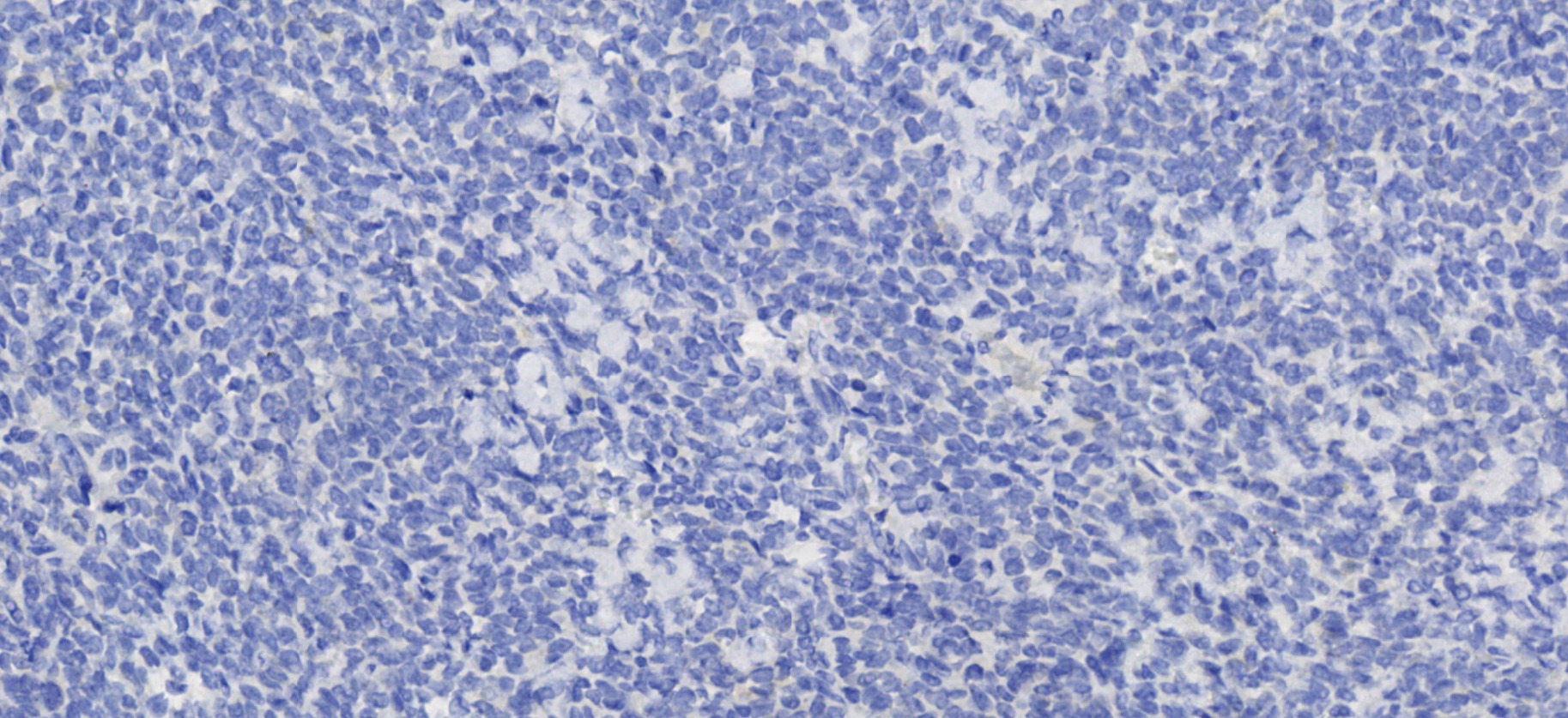

Supplement: Supplementary file 1 [file DataSheet_1.zip › Supplementary Materials/PD-1 Surgical specimens.jpg]

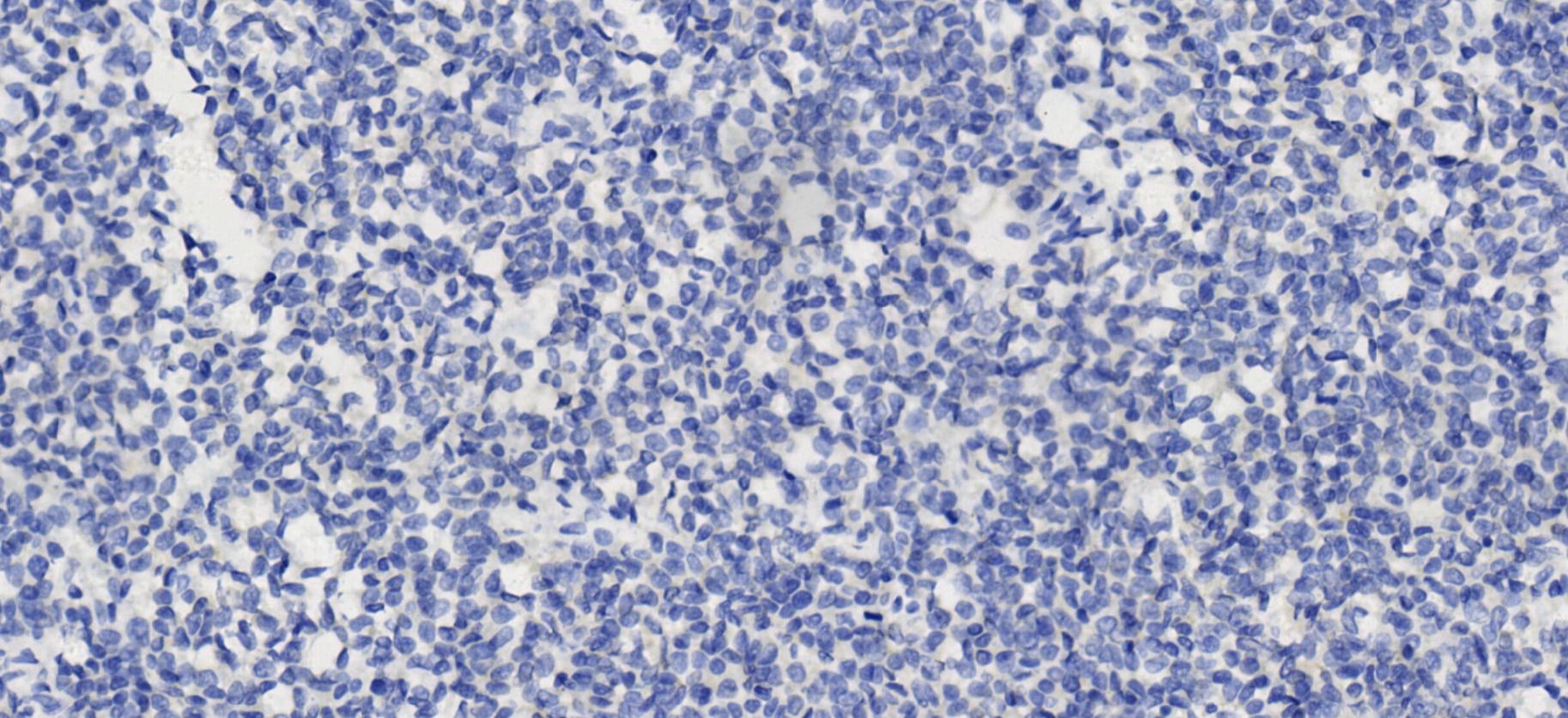

Supplement: Supplementary file 1 [file DataSheet_1.zip › Supplementary Materials/PD-L1 Surgical specimens.jpg]

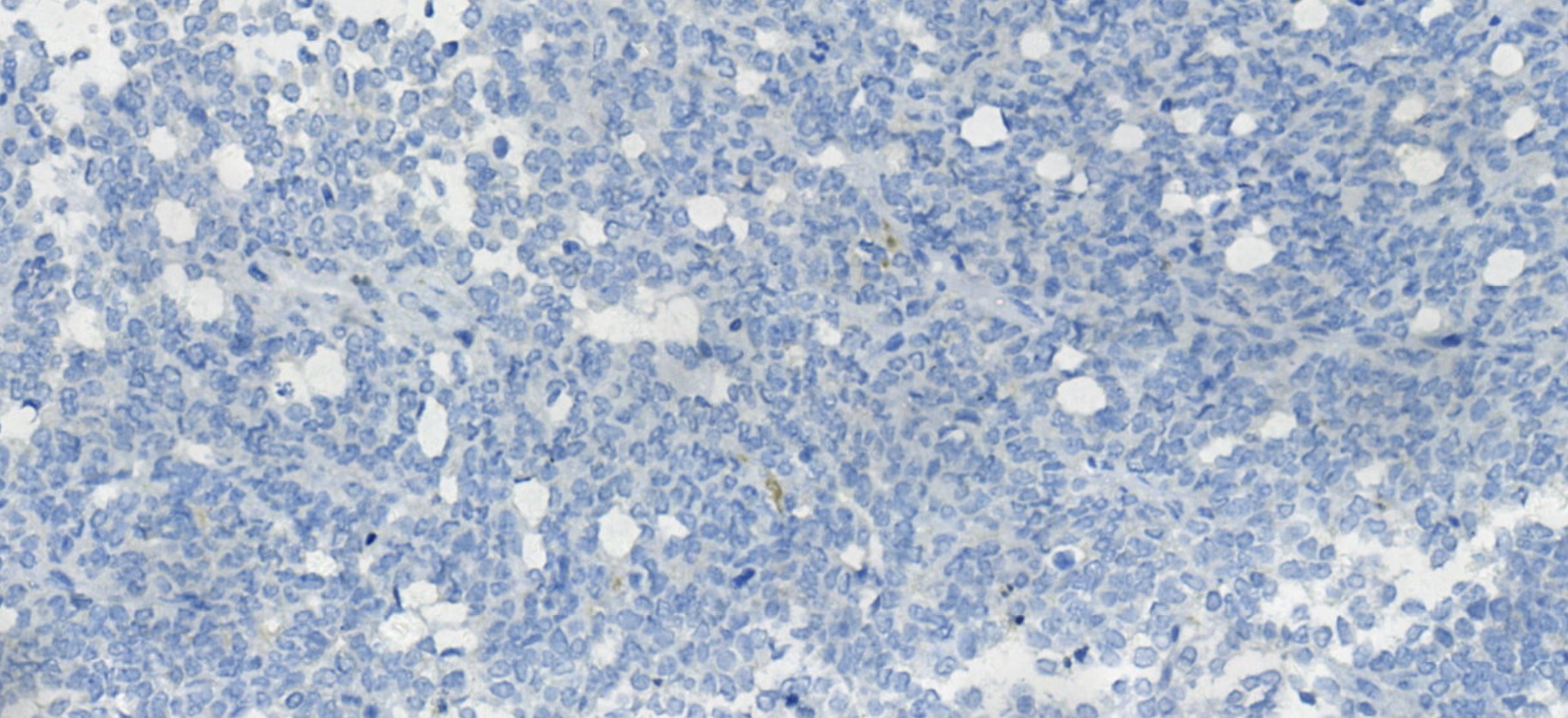

Supplement: Supplementary file 1 [file DataSheet_1.zip › Supplementary Materials/S100 biopsy specimen.jpg]

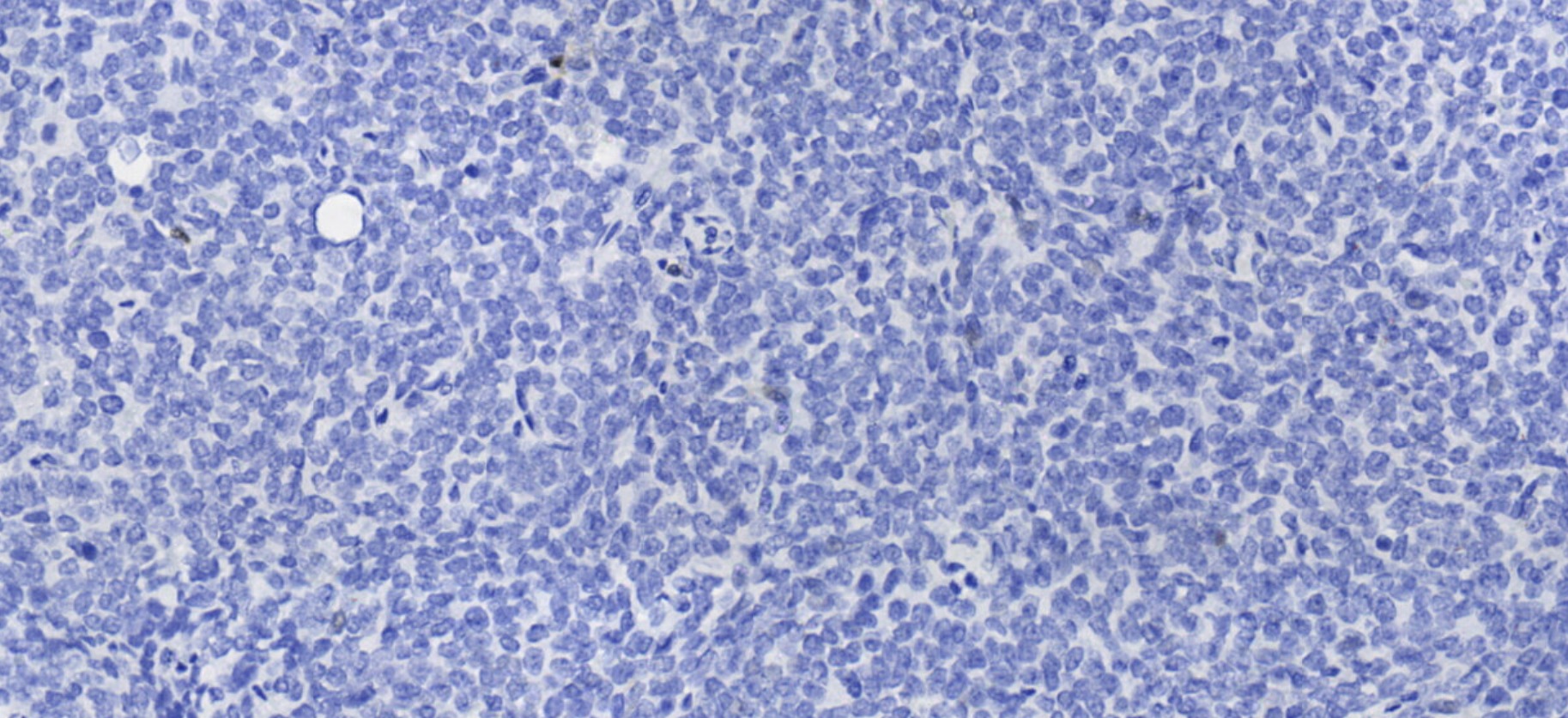

Supplement: Supplementary file 1 [file DataSheet_1.zip › Supplementary Materials/S100 Surgical specimens.jpg]

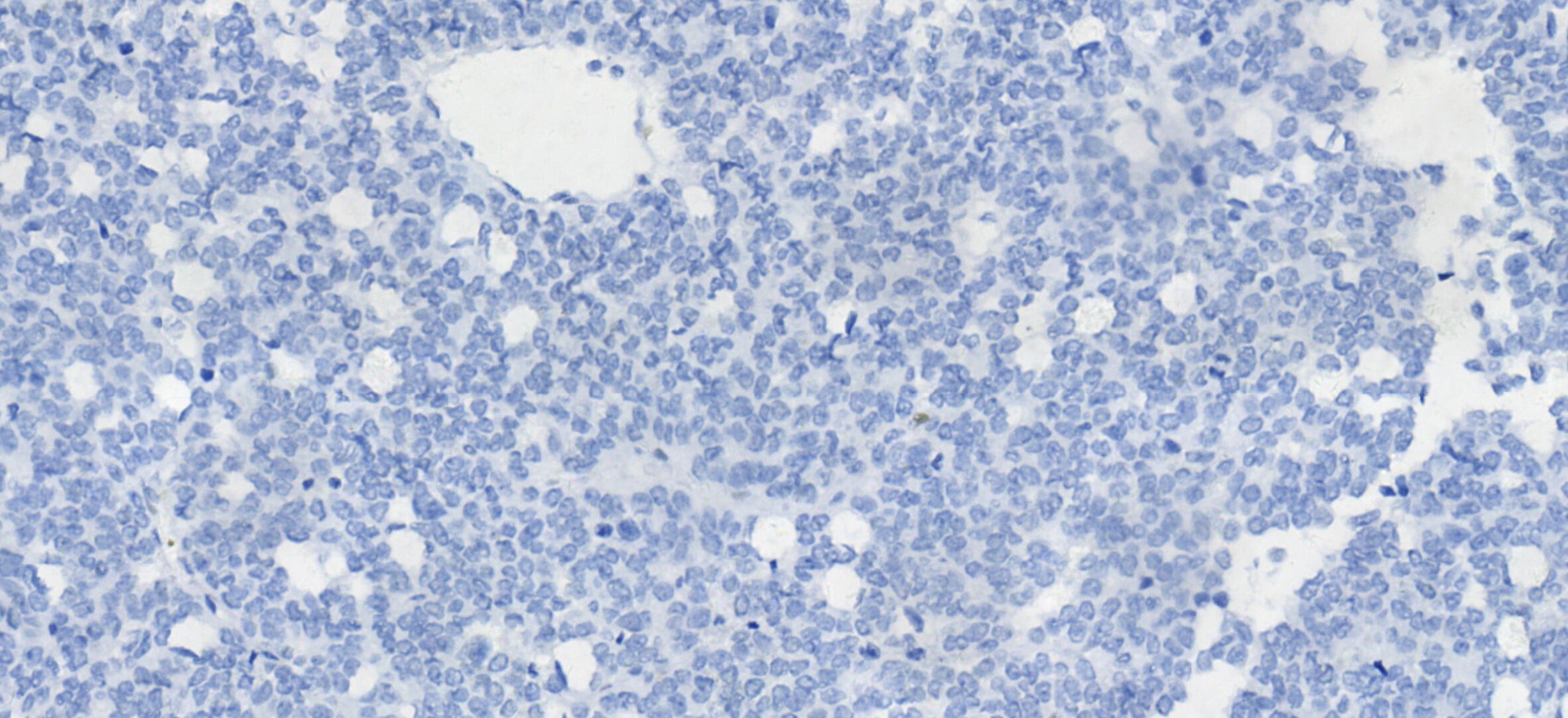

Supplement: Supplementary file 1 [file DataSheet_1.zip › Supplementary Materials/SATB2 biopsy specimen.jpg]

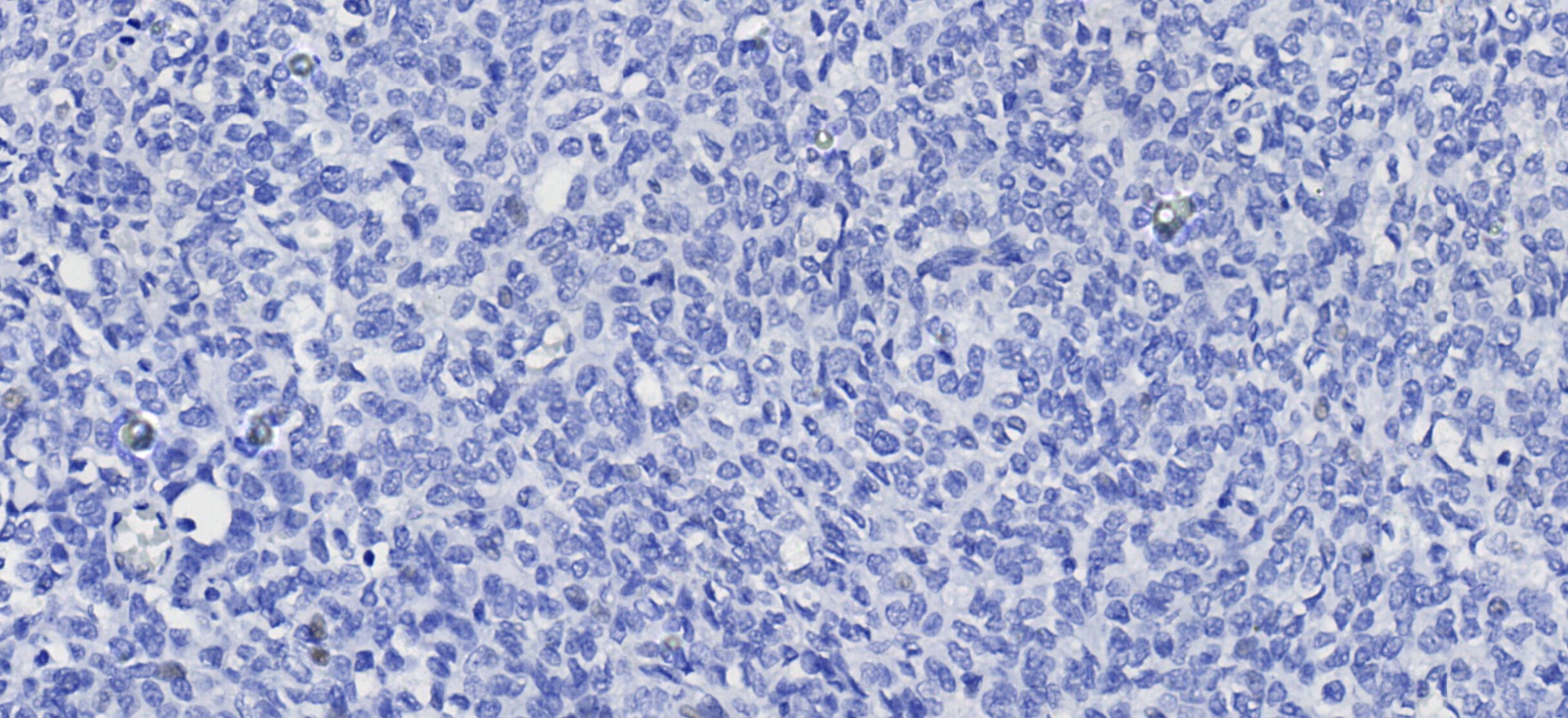

Supplement: Supplementary file 1 [file DataSheet_1.zip › Supplementary Materials/SATB2 Surgical specimens.jpg]

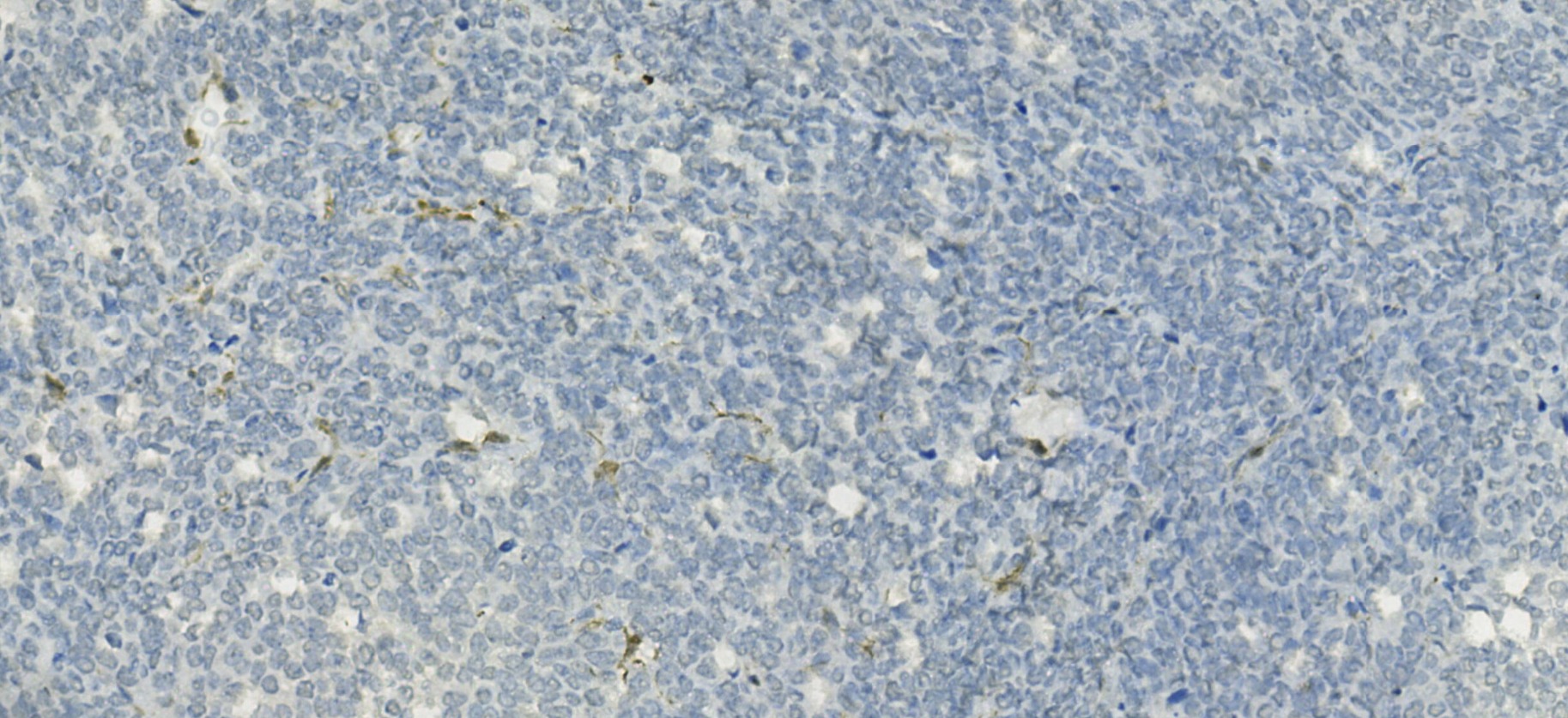

Supplement: Supplementary file 1 [file DataSheet_1.zip › Supplementary Materials/SMA biopsy specimen.jpg]

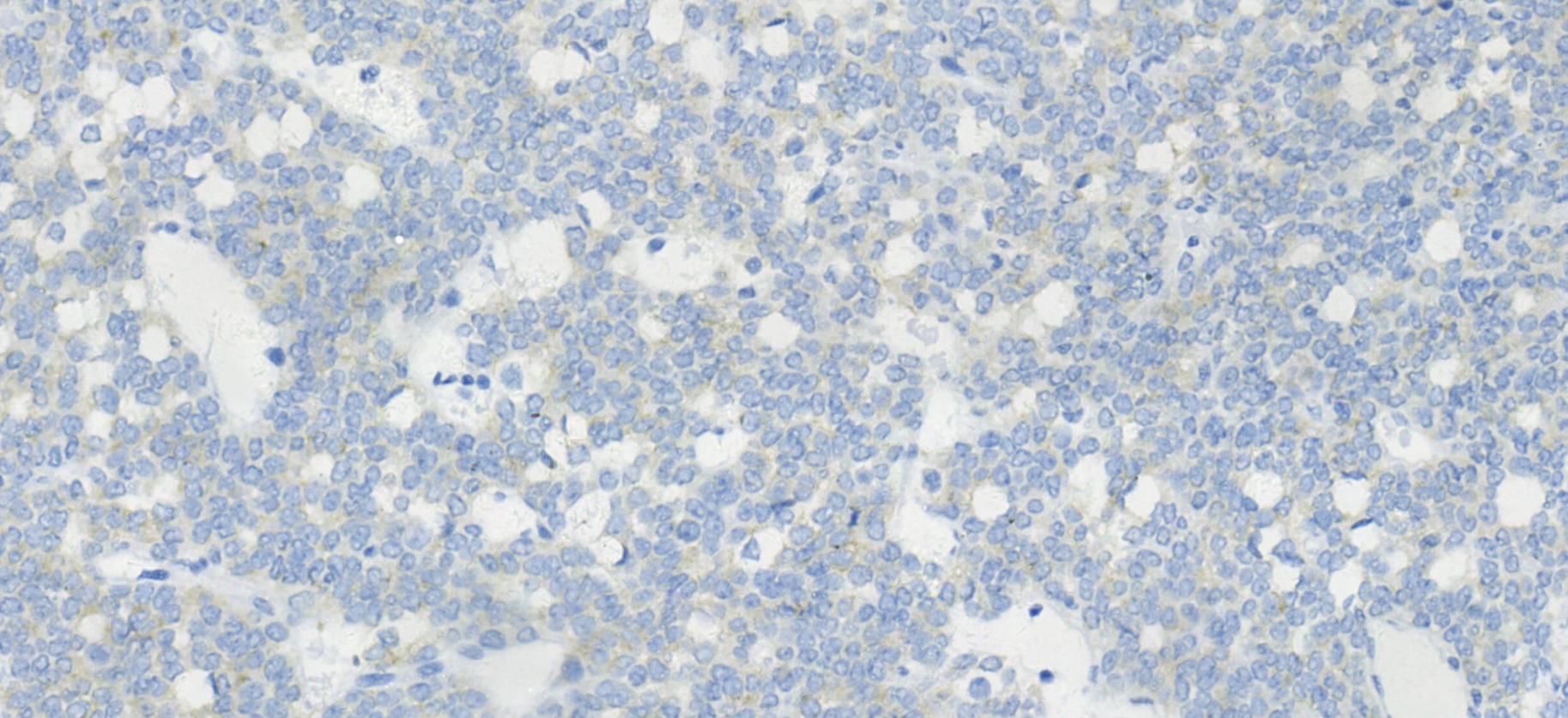

Supplement: Supplementary file 1 [file DataSheet_1.zip › Supplementary Materials/Syn biopsy specimen.jpg]

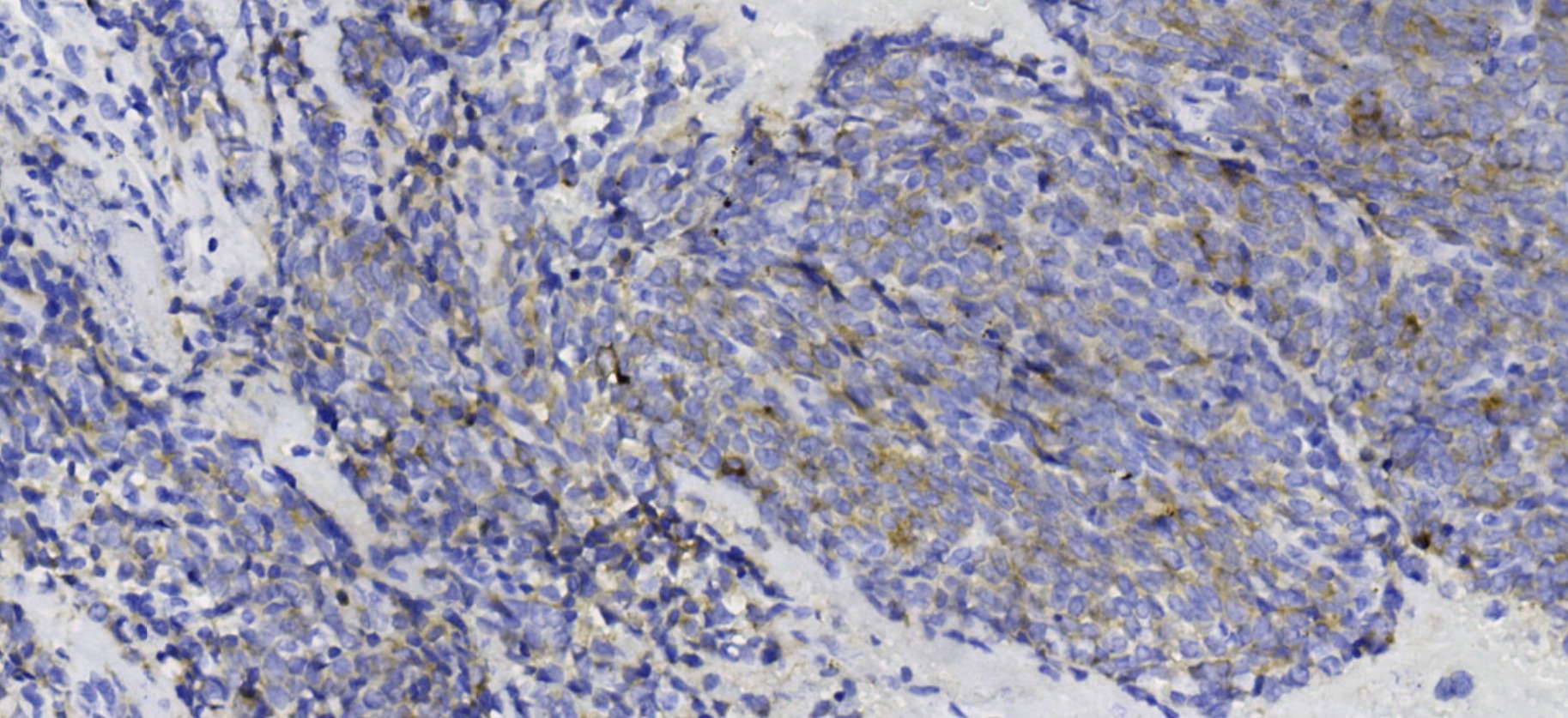

Supplement: Supplementary file 1 [file DataSheet_1.zip › Supplementary Materials/Syn-Surgical specimens.jpg]

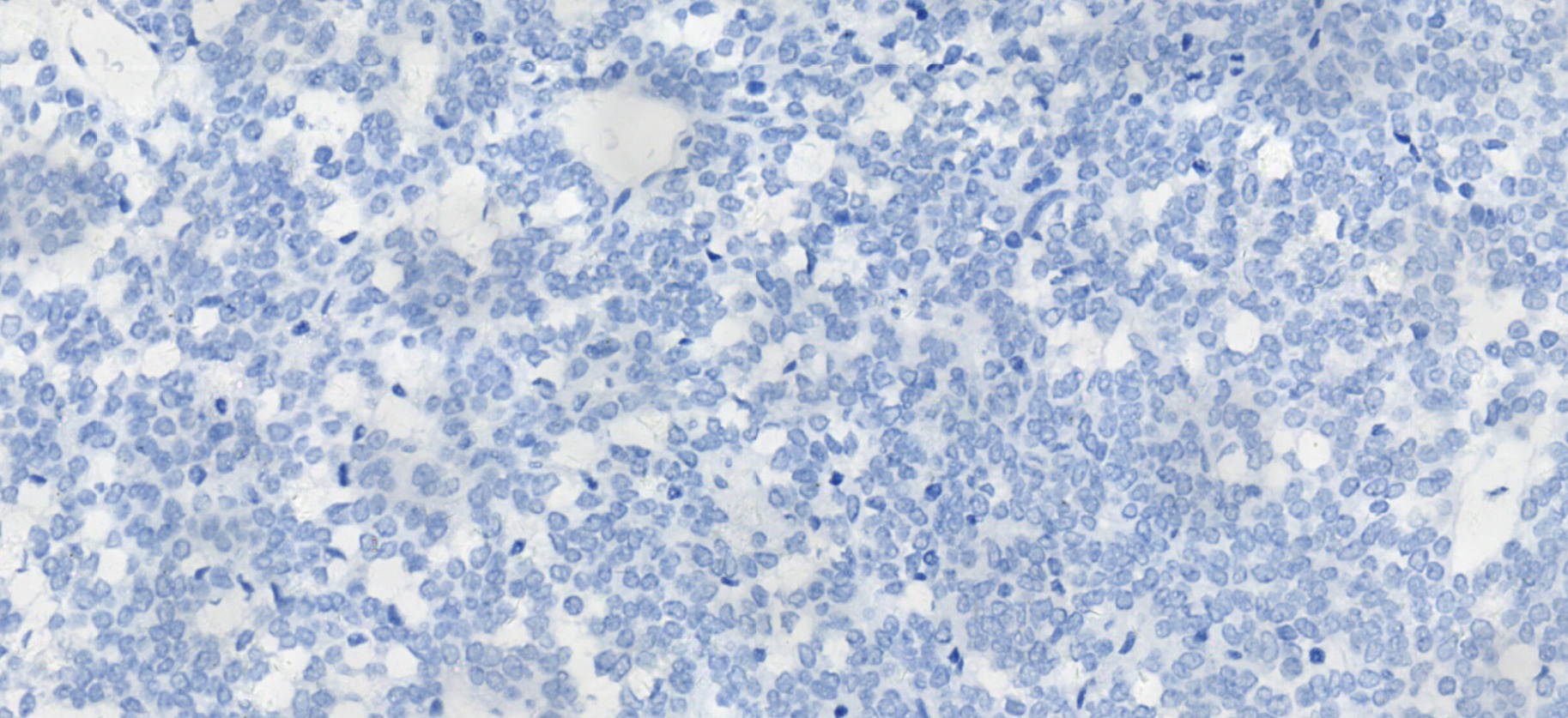

Supplement: Supplementary file 1 [file DataSheet_1.zip › Supplementary Materials/TTF-1 biopsy specimen.jpg]

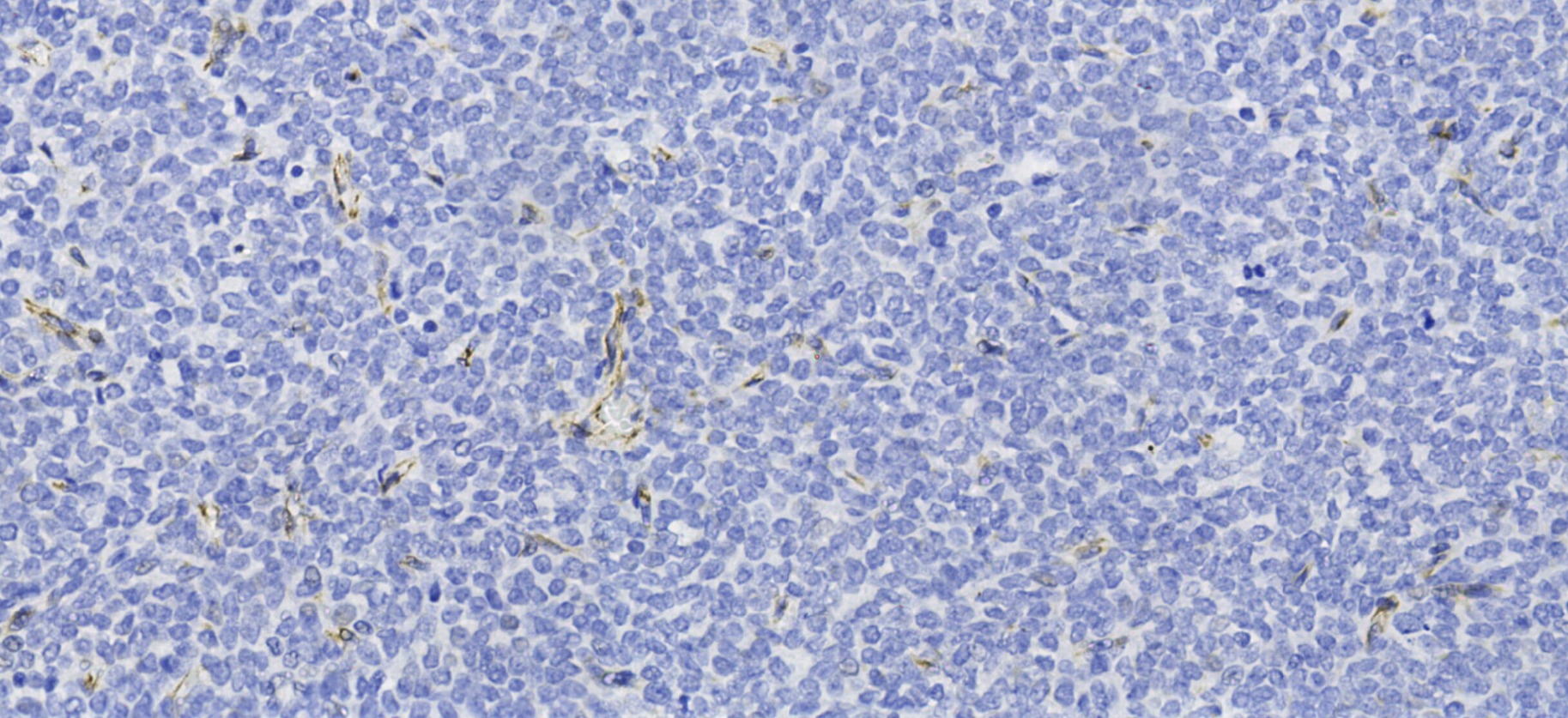

Supplement: Supplementary file 1 [file DataSheet_1.zip › Supplementary Materials/Vim Surgical specimens.jpg]

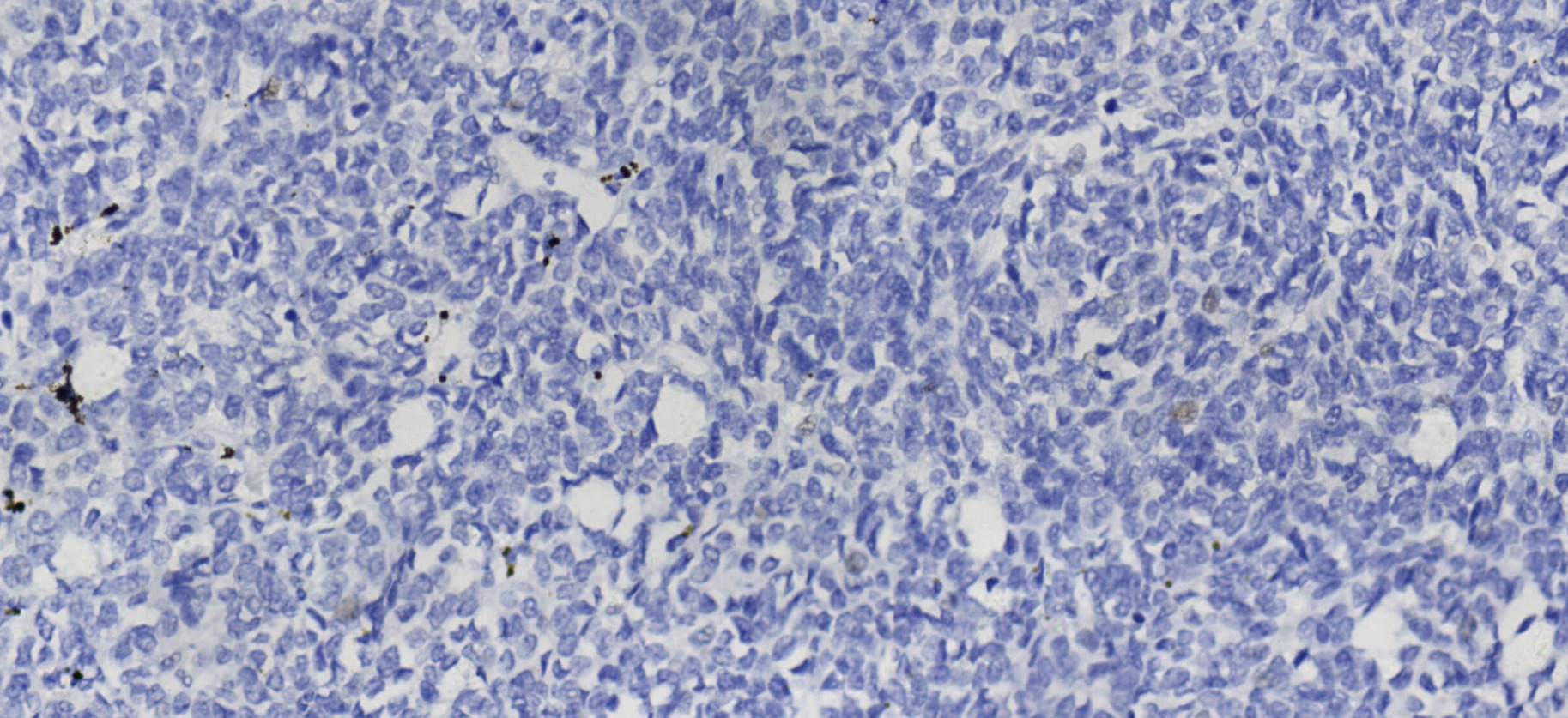

Supplement: Supplementary file 1 [file DataSheet_1.zip › Supplementary Materials/WT-1 Surgical specimens.jpg]
